# The Mechanochemistry of Endocytosis

**DOI:** 10.1371/journal.pbio.1000204

**Published:** 2009-09-29

**Authors:** Jian Liu, Yidi Sun, David G. Drubin, George F. Oster

**Affiliations:** Department of Molecular and Cell Biology, University of California Berkeley, Berkeley, California, United States of America; Princeton University, United States of America

## Abstract

Endocytic vesicle formation is a complex process that couples sequential protein recruitment and lipid modifications with dramatic shape transformations of the plasma membrane. Although individual molecular players have been studied intensively, how they all fit into a coherent picture of endocytosis remains unclear. That is, how the proper temporal and spatial coordination of endocytic events is achieved and what drives vesicle scission are not known. Drawing upon detailed knowledge from experiments in yeast, we develop the first integrated mechanochemical model that quantitatively recapitulates the temporal and spatial progression of endocytic events leading to vesicle scission. The central idea is that membrane curvature is coupled to the accompanying biochemical reactions. This coupling ensures that the process is robust and culminates in an interfacial force that pinches off the vesicle. Calculated phase diagrams reproduce endocytic mutant phenotypes observed in experiments and predict unique testable endocytic phenotypes in yeast and mammalian cells. The combination of experiments and theory in this work suggest a unified mechanism for endocytic vesicle formation across eukaryotes.

## Abstract 2

An integrated theoretical model reveals how the chemical and the mechanical aspects of endocytosis are coordinated coherently in yeast cells, driving progression through the endocytic pathway and ensuring efficient vesicle scission in vivo.

## Author Summary

Endocytosis is a complex and efficient process that cells utilize to take up nutrients and communicate with other cells. Eukaryotes have diverse endocytic pathways with two common features, mechanical and chemical. Proper mechanical forces are necessary to deform the plasma membrane and, eventually, pinch off the cargo-laden endocytic vesicles; and tightly regulated endocytic protein assembly and disassembly reactions drive the progression of endocytosis. Many experiments have yielded a lot of detailed information on the sub-processes of endocytosis, but how these sub-processes fit together into a coherent process in vivo is still not clear. To address this question, we constructed the first integrated theoretical model of endocytic vesicle formation, building on detailed knowledge from experiments in yeast. The key notion is that the mechanical force generation during endocytosis is both slave to, and master over, the accompanying endocytic reaction pathway, which is mediated by local membrane curvature. Our model can quantitatively recapitulate the endocytic events leading to vesicle scission in budding yeast and can explain key aspects of mammalian endocytosis. The phenotypes predicted from variations within the feedback components of our model reproduce observed mutant phenotypes, and we predict additional unique and testable endocytic phenotypes in yeast and mammalian cells. We further demonstrate that the functional significance of such mechanochemical feedback is to ensure the robustness of endocytic vesicle scission.

## Introduction

During clathrin-mediated endocytosis, cells regulate plasma membrane molecular composition and internalize essential nutrients. This process involves coordination of biochemical activities with membrane shape changes [1],[2]. Multicolor real-time fluorescence microscopy studies in mammalian cells and yeast established that proteins are sequentially recruited to the endocytic site to drive membrane invagination and vesicle scission [3],[4],[5],[6],[7],[8],[9],[10],[11],[12]. Real-time movies and EM studies in yeast and mammals have demonstrated that the endocytic membrane is composed of different regions (bud and tubule/neck), each with a distinct protein composition and spatial profile [13],[14],[15]. Comparisons between yeast and mammalian endocytic systems have highlighted similarities and differences [2],[16]. The extent to which common principles underlie endocytosis in different eukaryotic cells is currently a matter of speculation and debate. Among the most obvious differences, clathrin-mediated endocytosis in mammalian cells involves formation of spherical clathrin-coated vesicle buds and recruitment of the GTPase dynamin to the vesicle neck, while endocytic structures in yeast are tubular invaginations lacking dynamin [15],[17]. Also, actin assembly is required for formation of the membrane invagination and for vesicle scission in yeast [8], while in mammalian cells these steps appear only to be assisted by actin assembly [18]. On the other hand, many endocytic proteins, including clathrin, adaptor proteins, and cytoskeletal proteins, are highly conserved from yeast to mammals. In both yeast and mammalian cells, dynamics of the key endocytic proteins are coordinated in space and time, and internalization and vesicle scission are accompanied by a transient burst of actin assembly [1],[2]. Despite intensive study in many laboratories, the mechanisms underlying coordination of protein recruitment, lipid modification, and membrane shape changes are not well understood in any organism.

From a mechanical standpoint, endocytosis appears to proceed in two stages: invagination of the membrane, followed by pinching off of the vesicle. The cell cortex is quite resistant to deformation, so the shape changes accompanying endocytosis incur a large energy penalty [19]. Consequently, the cell must generate a considerable mechanical force to deform the endocytic membrane. To do so, endocytosis must involve biochemical reactions at the endocytic site that control the pulling and pinching forces. In budding yeast, actin polymerization and myosin motor activity have been implicated in providing the pulling force for membrane invagination [10]. Pinching off of the membrane vesicle entails even larger membrane curvatures at the scission site than does generation of the invaginated membrane. In mammalian cells, dynamin GTPases have been proposed to act as “pinchases” that physically constrict membrane tubules [20],[21]. However, endocytic vesicles form in budding yeast despite the absence of dynamin at endocytic sites. In vitro studies have suggested a possible scission mechanism; an interfacial force arising at the boundary between two lipid phases can provide the driving force for vesicle scission [22],[23]. We previously proposed that such a mechanism might drive endocytic vesicle scission in vivo [11],[24].

Reciprocally, emerging experimental evidence suggests that membrane curvature created by mechanical force can modulate the local biochemical activities of several key endocytic proteins [25]. Experiments suggest that membrane curvature may act as a guiding signal to direct BAR (Bin/Amphiphysin/Rvs) domain proteins to the endocytic membrane invagination [26]. Conversely, BAR domain proteins (BDPs) are also capable of deforming the membrane into the preferred shape for their binding [27],[28],[29],[30],[31],[32]. However, in the context of the coherent process of endocytosis, the exact functional role of these physical properties of endocytic proteins remains elusive.

Here we attempt to combine detailed knowledge of endocytic protein dynamics and function in budding yeast with mechanochemical concepts to develop an integrated systems model for the endocytic internalization pathway. Our model stands in contrast to previous models [22],[23],[24]. Rather than focusing on one sub-process, our model seeks to reproduce the correct sequence of events in a coherent manner, including the local biochemical reactions and membrane shape changes. We propose a mechanochemical feedback mechanism that can generate successful endocytosis over a broad range of its parameter space. The model fits quantitatively the correct temporal and spatial profiles measured in budding yeast. Furthermore, when the parameters are varied to mimic endocytic mutants, the model accounts for many endocytic phenotypes in budding yeast and yields experimentally testable predictions. Finally, we argue that, despite some differences in molecular details, the underlying principles likely apply to mammalian endocytosis as well.

## Results

### Qualitative Description of Model

In this section, we will describe the qualitative features of our model. The quantitative mathematical formulations will be relegated to the Experimental Procedures.

**Figure 1:**
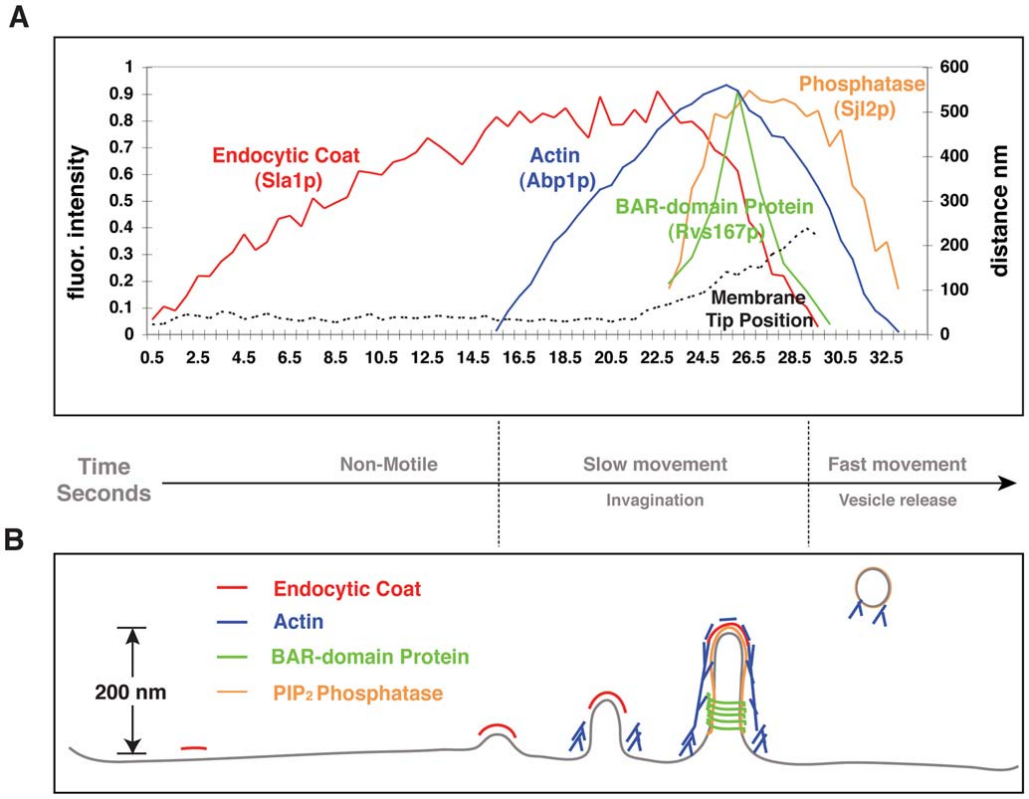
Endocytic dynamics in budding yeast. (A) Timelines for endocytic protein recruitment as determined by multicolor fluorescence microscopy analysis. Sla1p, which is an endocytic adaptor protein, represents the endocytic coat. Abp1p is an actin-binding protein and faithfully reports on actin dynamics. Sjl2p is the yeast synaptojanin that hydrolyzes PIP_2_. PIP_2_ represents the lipid module and is believed to be the recruitment signal for many endocytic proteins. Rvs167p, yeast Amphiphysin, contains a BAR domain capable of sensing/binding curved membranes and deforming membranes. (Sla1 and Abp1 data are from [8], Sjl2 data are from [11], Rvs167 data are determined in this work from six individual patches in cells expressing Rvs167-GFP and aligned to the relative timing of Sjl2 appearance.) (B) Spatial profiles of endocytic membrane and the key endocytic proteins as revealed by EM [15].

Temporal control and spatial arrangement of proteins and the lipid PI(4,5)P_2_ at budding yeast endocytic sites are key features in the development of our model (Figure 1). First, each of the key endocytic proteins appears to localize along the membrane invagination with a distinct spatial profile, predicted by dynamic properties [8],[9],[11] and confirmed by EM [15]. Second, these proteins can be grouped into four “protein modules” based on their distinct dynamics and functions [9],[15]. Lastly, we previously obtained evidence for a PI(4,5)P_2_ “lipid module” that is dynamically regulated during endocytosis [11].

**Figure 2:**
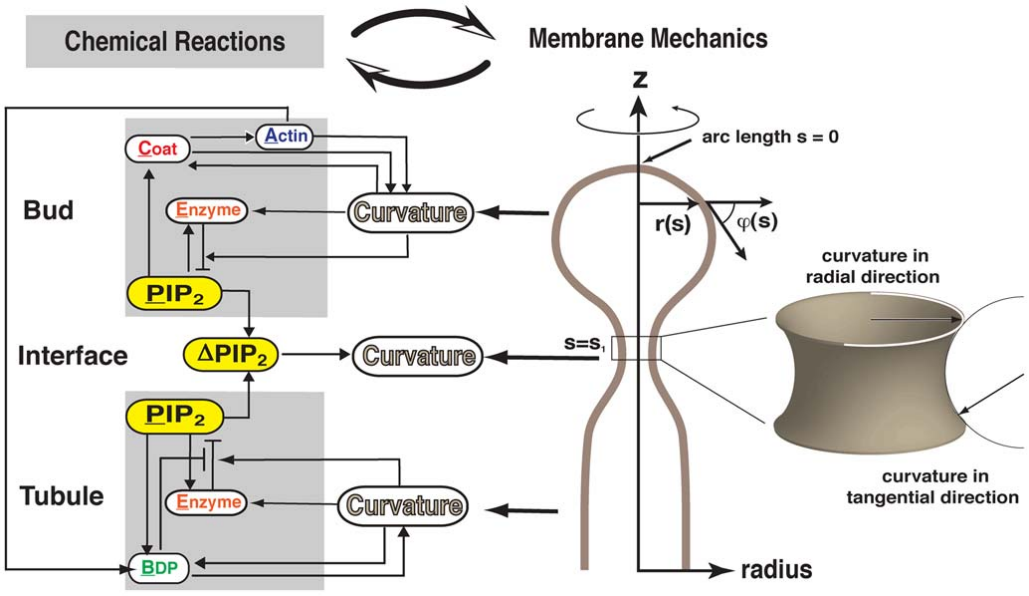
Mechanochemical feedback mechanism for endocytosis in budding yeast. The thin arrows represent activation effects, and the bar ends represent inhibition effects. The local spatial coordinate along the membrane surface is the arc length *s* with unit length 1 nm. The bud region is defined by the arc length 0≤*s*≤*s*_1_, the lipid phase boundary is at *s* = *s*_1_, and the tubule region starts from *s* = *s*_1_+1, where *s*_1_ is chosen to be 100. We assume that membrane shape is cylindrically symmetric. *ϕ*(*s*) is the membrane tangential angle and *r*(*s*) is the radius of the tubule. The mean curvature, Ω(s), is the average of the curvatures in the radial and tangential directions; it measures the overall extent of the PIP_2_ head group exposure.

For this model, we describe clathrin-mediated endocytic dynamics on the level of functional modules, which allows us to look beyond roles of individual molecular players that may vary from one organism to another and to focus upon collective behaviors in membrane shape transformations and local biochemical pathways. Thus, our model can serve as a unified framework for endocytosis across diverse organisms. We propose that the five modules along with their functions are as follows (Figure 2 provides an overview of the model):

1. Phosphoinositides, e.g., PI(4,5)P_2_ (PIP_2_), cover the endocytic membrane and recruit endocytic proteins to the plasma membrane [11],[15],[33],[34]. PIP_2_ accumulation driven by lipid kinases, and its hydrolysis by phosphatases, proceeds at the endocytic membrane throughout the course of endocytosis [11],[34],[35],[36],[37],[38]; local PIP_2_ levels are controlled by the balance between accumulation and hydrolysis.
2. Coat proteins (e.g., clathrin and Sla1) accumulate on the vesicle bud via interaction with PIP_2_ or PIP_2_-associated adaptor proteins [8],[12],[33],[39],[40],[41],[42]. The coat proteins anchor and regulate actin filaments while imparting curvature to the bud region [43].
3. Proteins that accumulate in the tubule region, e.g., BDPs [9],[15],[44], have both membrane-deforming and membrane curvature–sensing power [25]. Taking into account the specific spatial and temporal profile of BDPs during endocytosis [9] and their binding to PIP_2_ (Kishimoto and Drubin, unpublished results), we further propose that BDPs generate a lipid phase boundary by protecting the underlying PIP_2_ from hydrolysis by the phosphatase, as suggested by experiments showing PIP_2_ clustering by BDPs [45].
4. The actin module proteins are anchored to the bud by binding to coat proteins. Actin and actin-associated proteins (i.e., F-actin and myosin) are responsible for generating the pulling force exerted on the bud [3],[4],[8],[9],[10]. The pulling force helps to generate BDP-binding sites, helping to recruit BDPs to the endocytic site [9].
5. Enzymes that hydrolyze PIP_2_, e.g., synaptojanin or Sjl2p in yeast [11],[35],[36],[37],[38], accumulate late in the vesicle formation process. In vitro experiments show that phosphoinositide hydrolysis rates by phospholipase C critically depend on the local membrane curvature [46]. Here we refer to the mean membrane curvature, which is the average of the curvatures in the tangential and radial directions on the membrane (see Figure 2). The mean curvature represents the extent of lipid head group exposure. At higher membrane curvatures, the enzymes have greater access to the lipid head groups, which enhances both the binding to the lipids and the enzyme's hydrolysis activity (see Protocol S1 for details). This curvature sensitivity of enzyme activities may be a general phenomenon, as suggested by the observation that PI3K kinase activity also critically depends on membrane curvature [47]. We propose, therefore, that a similar mechanism applies to PIP_2_ hydrolysis, which has been corroborated by experiments (Chang-Ileto and Di Paolo, personal communication).

From a mechanical standpoint, the pulling forces generated by the actin/myosin functional module impinge on the bud and invaginate the membrane. The initial pinching force is generated as follows. Because of the protection afforded by BDPs on the tubule, more PIP_2_ is hydrolyzed at the bud region. This leads to lipid phase segregation—PIP_2_ levels along the membrane invagination differ, and the resulting interfacial force at the bud-tubule interface squeezes the neck. From a chemical perspective, the local chemical reactions (e.g., actin assembly, PIP_2_ hydrolysis) control pulling and pinching forces. Equally important, the resulting membrane curvature generated by the mechanical forces also influences the local reaction rates (Figure 2). In this way, endocytic dynamics are controlled by mechanochemical feedback between endocytic membrane shape changes (membrane curvature) and the local chemical reactions that control the mechanical forces (pulling and pinching forces). This key notion, as we will show below, is essential for the robustness of the sequential endocytic protein recruitment and timely vesicle scission.

This qualitative picture is captured by Equations 1–6 in the Experimental Procedures. The coupling between the mechanical and chemical processes of endocytosis is specified by the dependence of the reaction rates on membrane curvature and by the dependence of the local membrane curvature and the mechanical force on the local levels and activities of the functional modules. To calculate the dynamics of endocytic events, we numerically integrate Equations 1–6 over time starting from the initial condition: the endocytic membrane is flat and the initial coverage for all of the protein modules is set to zero. The initial PIP_2_ coverage is set to 2% corresponding to its normal average level [48]. At each step, the system is characterized by the instantaneous shape of the endocytic membrane and the local levels of the functional modules as represented in mole fraction. The values of the parameters used in the model are listed in Table 1 with references in Protocol S1. Below, we first study the endocytic dynamics of budding yeast by choosing the parameter set that quantitatively fits the time-lapse experimental data in Figure 1A. We then vary the parameters to mimic mutant experiments to predict and analyze the associated phenotypes.

As the model dynamics are controlled by many parameters in Equations 1–6, there could in principle be many outcomes depending on parameter choices. To circumvent this problem, 21 of the 25 parameters used in the model were taken from independent experiments (Table 1 in Protocol S1). The four unmeasured parameters all characterize BDP dynamics; they are the intrinsic BDP recruitment rate, actin-aided recruitment rate, turnover rate, and the relative timescale of BDP dynamics with regard to actin dynamics. With 21 measured parameters being fixed, we only vary the four free value parameters to fit the five time-lapse curves of endocytic dynamics observed in wild-type budding yeast (Figure 1A). The values of these four parameters are constrained because these kinetic rates must be comparable to those experimentally determined for each of the other functional modules. The dynamics of all of the modules are tightly coupled: one sub-process cannot be much faster/slower than the others. In what follows, we use specific proteins or lipids to represent the corresponding functional modules. We stress from outset that the goal of the paper is to illuminate the collective dynamics of endocytosis generated by the interactions among the functional modules, rather than identifying detailed molecular players.

### Endocytosis Involves a Precisely Timed and Ordered Sequence of Events

**Figure 3 pbio-1000204-g003:**
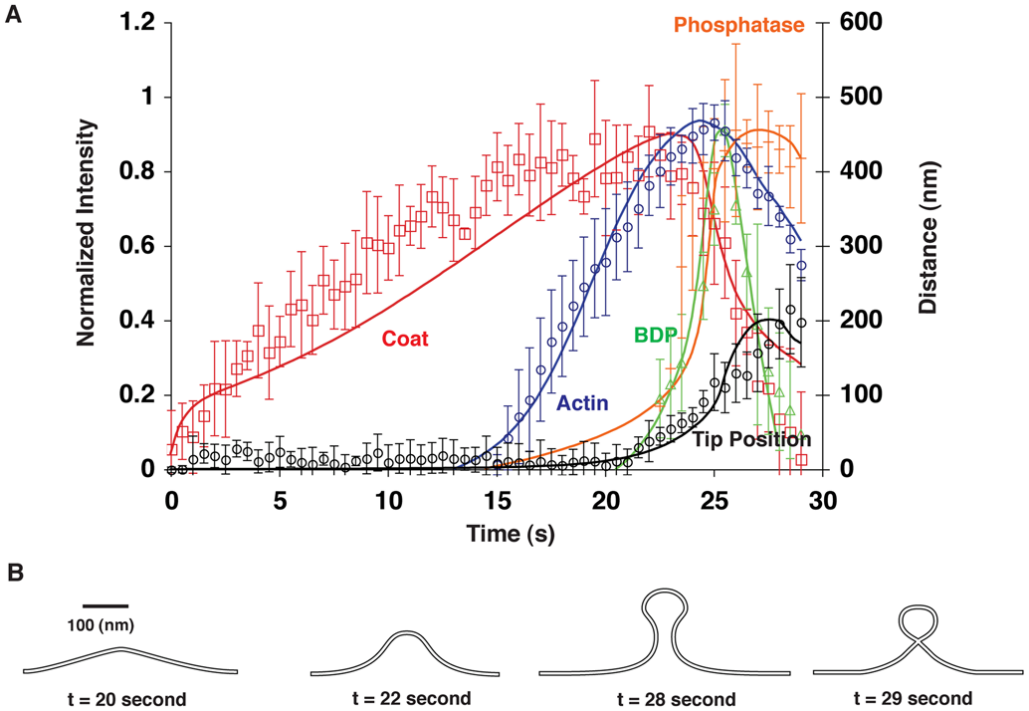
Fitting of the results calculated from the model to experimental results. (A) Timelines of functional modules during endocytosis in budding yeast (continuous lines represent calculated values, and the discontinuous lines are experimental measurements—same as Figure 1A—with standard deviation). In the model, the instantaneous total levels for the individual modules (except for actin) at the endocytic site were obtained by summing their local levels over their respective locations on the membrane surface. The instantaneous total level of actin was obtained by summing over the entire bud region the product of the local actin level and its distance from cell cortex (proportional to the length of the actin filaments). To obtain the intensity plot for each of the modules, we normalized the curve for its total levels over time in accordance to its respective peak value. We then scaled the resulting curve by setting its peak value to be the same as that of the peak intensity measured experimentally. We thus can compare the computed time-lapse curve for each module to those from experimental observations. (B) Calculated endocytic membrane shape changes. The calculation of membrane shape was carried out in 3-D. Membrane shape is shown in 2-D for clarity. The parameters in the model used for curve fitting are listed in Table 1 in Protocol S1. If not stated otherwise, the parameters are fixed throughout this paper.

Figure 3A shows that the endocytic dynamics predicted by our model (continuous lines) fit quantitatively with the experimental data (discontinuous lines) [8],[11, and the measurements in this paper]. Figure 3B shows snap shots of the corresponding computed membrane shape changes (a movie of the process derived from model calculation is provided in Video S1). Because the fitting parameters are constrained by measurements from independent studies, the agreement between our theoretical results and experimental observations lends validation to our model. An important feature of the process is that each functional module is activated sequentially in step with the membrane shape changes (Figure 3A and 3B). We next describe steps in the endocytic process in greater detail based on our model.

Early in the process (0–20 s, Figure 3A) coat proteins begin to accumulate. During this period, the membrane is deformed by the coat proteins, which generate a small dome (less than 50 nm in height and ∼50 nm in width, t∼20 s in Figure 3B). However, there is a delay before actin polymerization fully commences, because it takes a while for the nucleation factors to be recruited and activated and because actin assembly is autocatalytic due to Arp2/3 activation by actin filaments. Without the assistance of the actomyosin force, the dome-like membrane deformation would not progress further, which is consistent with observations from recent EM studies [15]. Indeed, this dome shape could be the prerequisite for further development of a deep invagination, because the local membrane shape may provide a suitable angle at which the F-actin pulling force can be exerted upon the bud region effectively.

At ∼20–25 s (Figure 3A), F-actin polymerization is promoted by nucleation factors recruited by the coat proteins, and the pulling force upon the bud region increases. This drives the endocytic membrane to invaginate further (t∼22 s in Figure 3B). As the membrane invaginates, actin monomers rapidly incorporate into the existing actin filaments with their barbed ends facing the cell cortex [8], while myosin pushes the actin network away from the plasma membrane into the cytoplasm. Meanwhile, the PIP_2_ phosphatase begins to accumulate all over the endocytic site. Concurrently, BDPs also start to accumulate along the tubule region rapidly, and they increase from 10% to the peak level in only 3 s (Figure 3A).

**Figure 4 pbio-1000204-g004:**
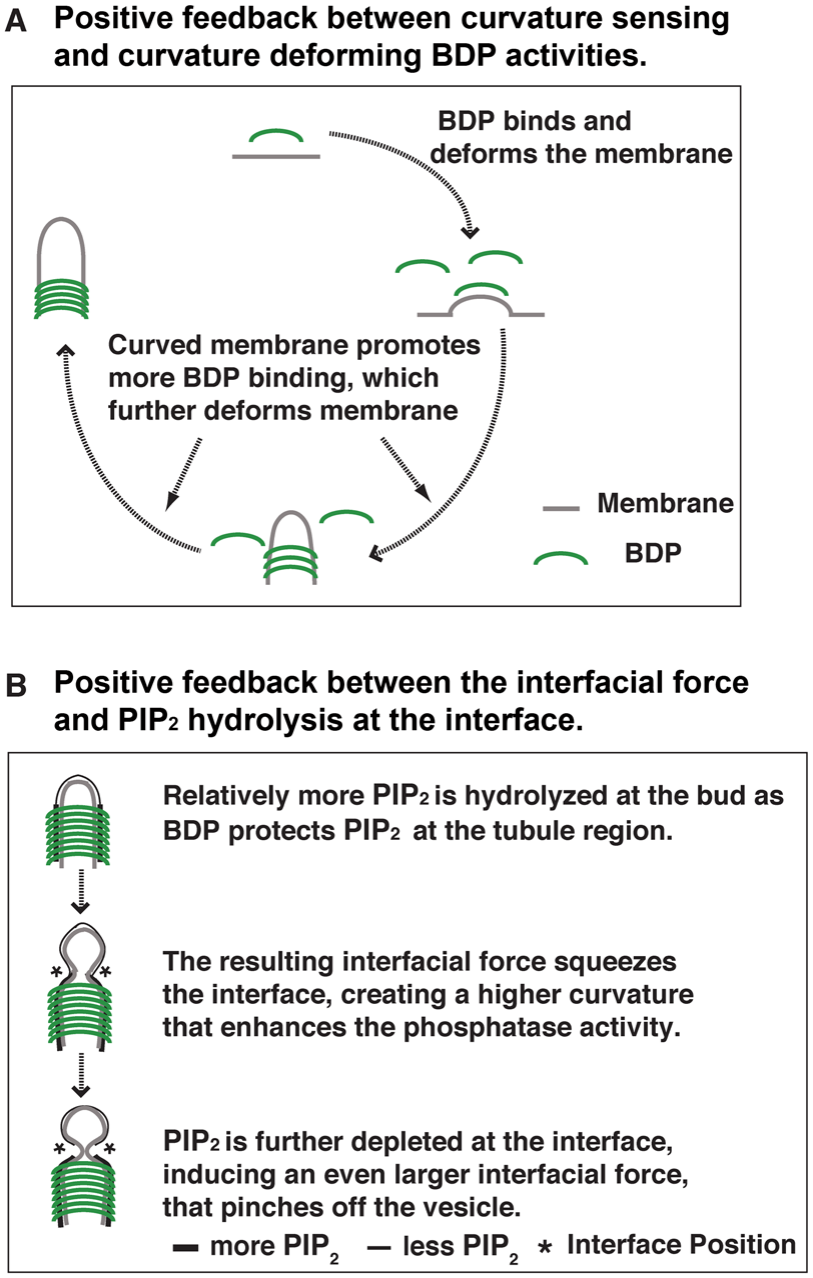
Two positive mechanochemical feedback loops between membrane shape changes and local chemical reactions. (A) Membrane tubulation by BDPs. (B) Development of interfacial forces that drive vesicle scission.

Now the question is: what drives the rapid BDP accumulation? We show that curvature-sensing and deforming activities of BDPs form an intrinsic positive feedback loop (see quantitative calculations in Figure S1). As schematized in Figure 4A, as they bind to the membrane, BDPs deform the adjacent membrane into the preferred curvature for their binding. This leads to a faster recruitment rate, which further promotes BDP recruitment and tubulation of the membrane. This positive feedback also explains and reconciles the two classes of experimental observations, which provided evidence for curvature-sensing and membrane-deforming activities [25],[26],[27],[28],[29],[30],[31],[32]. In our scenario, actin assembly and myosin contractile forces invaginate the membrane. The resulting membrane curvature fits relatively favorably to the preferred shape of BDPs and, hence, promotes rapid BDP binding at the right location and at the right time due to the curvature-sensing activity. In turn, BDP binding invaginates the membrane further and generates optimal curvature for BDP binding in the elongating tubule, which self-accelerates BDP accumulation. Thus, the initial membrane invagination generated by the actin/myosin force triggers the positive feedback between BDP binding and membrane tubulation.

During this same period, PIP_2_ hydrolysis rates are faster on the bud than on the tubule, as the BDPs protect the PIP_2_ on the underlying tubule from hydrolysis. Lipid-protein interactions involving BDPs could limit PIP_2_ diffusion in the membrane [45], allowing formation of a lipid-phase boundary. An interfacial force at the bud-tubule boundary thus starts to build up, constricting the neck.

Eventually (t∼29 s in Figure 3A and 3B), the interfacial force narrows the neck down to <5 nm, at which distance the opposed bilayers would fuse spontaneously [49], resulting in rapid vesicle scission. Upon vesicle scission, BDPs disassemble from the membrane tubule within 3 s as the tubule retracts due to loss of the actin pulling force. A second crucial effect of the PIP_2_ phosphatase activity on the vesicle bud is to trigger disassembly of the endocytic coat (t∼25–29 s, Figure 3A). As coat proteins disassemble, the F-actin attachments to the bud weaken, resulting in loss of pulling force on the invagination. We predict that this leads to a small retraction of the endocytic membrane tip concurrent with vesicle scission (see Figure 3A) and propose that loss of the pulling force on the membrane may be a prerequisite for vesicle scission.

### Rapid Vesicle Scission Is Triggered by Lipid Phase Segregation via Curvature-Enhanced PIP_2_ Hydrolysis

**Figure 5 pbio-1000204-g005:**
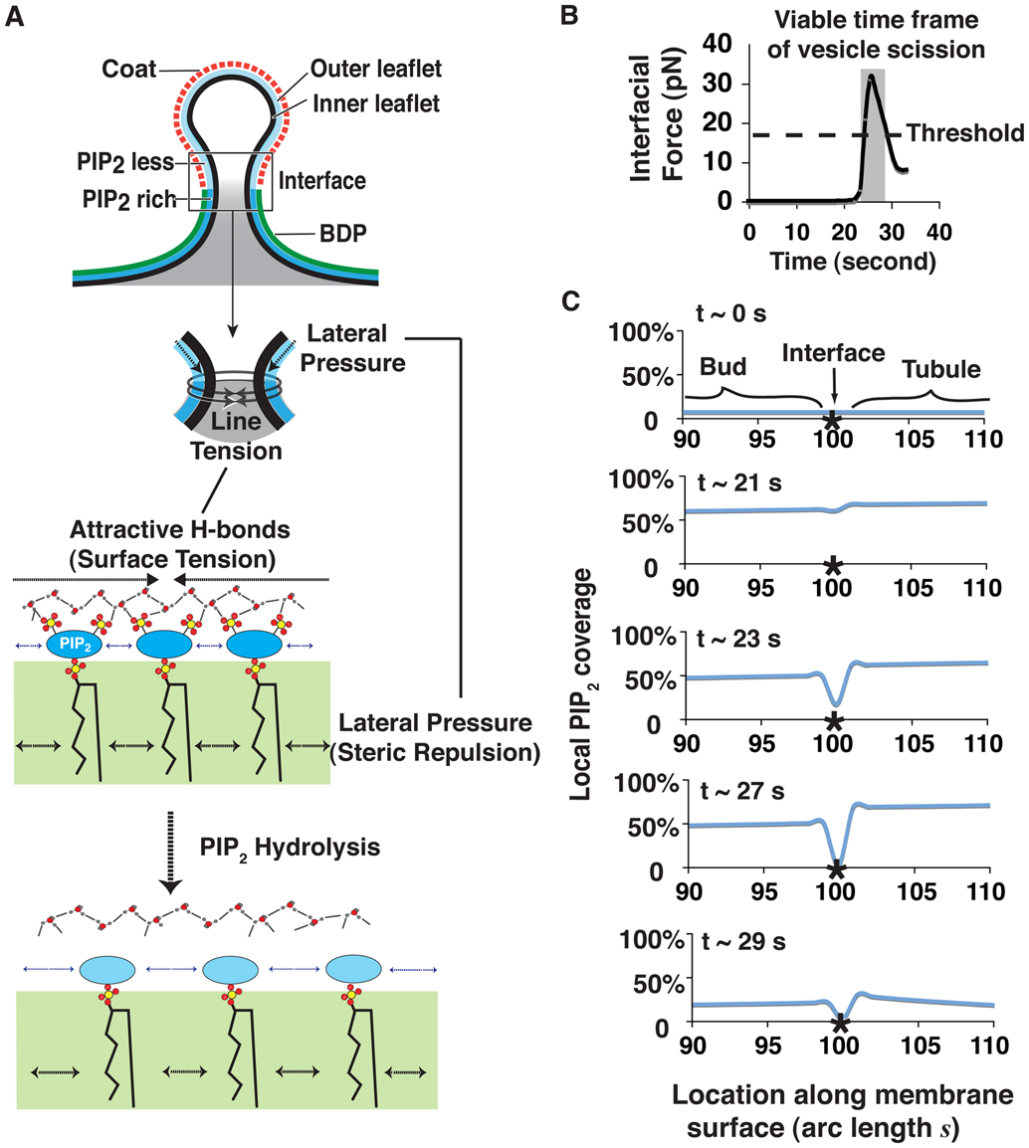
Development of the interfacial force during endocytosis. (A) Schematics of interfacial forces that consist of two components. The first is the line tension. Because less PIP_2_ is hydrolyzed on the tubule, a higher hydrogen bond density is created adjacent to the bud. The imbalance in electrostatic attraction from hydrogen bonds between the two adjacent regions (bud and tubule) results in a line tension encircling the neck. The second force is the lateral pressure in the cytoplasmic leaflet of the bud membrane. The average area per PIP_2_ in the membrane is determined by the force balance between steric repulsion (i.e., arising from both the hydrocarbon chain and the polar head groups) and attractive electrostatic interactions (e.g., hydrogen bonds). The net effect of PIP_2_ hydrolysis is to decrease the electrostatic attraction more than the steric repulsion, causing the PIP_2_ leaflet to expand [56],[57]. The osmotic pressure in the cell inhibits the expansion in the normal direction, and so the cytoplasmic leaflet expands tangentially. (B) The calculated time course of the interfacial force. The threshold value for the interfacial force was determined by a force-balance calculation similar to [24]. (C) The computed time course for PIP_2_ levels around the lipid phase boundary (at the arc length *s* = 100).

Our description of the endocytic process (Figure 3) raises the following interesting questions: How is the interfacial scission force generated? How does vesicle scission occur so rapidly? And what turns off the positive feedback loop for BDP assembly and drives their extremely fast disassembly? In this section, we propose answers to these questions. Our proposal that an interfacial force can drive vesicle scission is supported by in vitro experiments [22],[23], in which lipid phase segregation is induced by lowering temperature. In vivo, however, cells always maintain constant temperature; instead, lipid-protein interactions could be utilized to yield effective lipid phase segregation. Here, we present two possible scenarios for how the interfacial force is developed in endocytosis (schematized in Figure 5A): (1) As PIP_2_ hydrolysis at the bud eliminates hydrogen bonds that had bridged the interfacial boundary, hydrogen bond shielding of the hydrophobic hydrocarbon chains is lost, and at the boundary these aliphatic tails are exposed to water, which is energetically unfavorable. The resulting line tension is proportional to the PIP_2_ difference across the interface, which contracts to minimize these unfavorable contacts, thus squeezing the neck. (2) The reduced hydrogen bond network at the bud lowers the membrane surface tension of the outer leaflet, which thus tends to expand. Effectively, this is a lateral surface pressure that propagates from the high-lateral pressure region towards the interfacial boundary. Due to the local concavity of the membrane created by the initial interfacial tension, this lateral pressure is directed inwards at the phase boundary and provides an additional pinching force. This additional lateral pressure also increases with the difference in PIP_2_ levels across the phase boundary (see the detailed derivations in Protocol S1).

Figure 5B shows the calculated time course for interfacial force development during endocytosis, while Figure 5C shows the calculated profiles for PIP_2_ levels around the bud-tubule boundary at different time points. Figure 5B and 5C show that the interfacial force undergoes rapid changes. During t∼0–21 s, PIP_2_ accumulates uniformly over the entire endocytic site, as promoted by kinase-mediated synthesis. From around t∼21 s (Figure 5C), PIP_2_ levels decline non-uniformly; consequently, the interfacial force starts to build up (Figure 5B). This spatial non-uniformity is because around the same time as the phosphatase is recruited, BDPs start to accumulate at the tubule region of the endocytic membrane (t∼21 s in Figure 3A). As a result of the BDP protection at the tubule, relatively more PIP_2_ is hydrolyzed on the bud, leading to lipid phase segregation at the BDP–coat protein boundary. This phase separation gives rise to the initial interfacial force at the phase boundary.

From t∼21–27 s (Figure 5B and 5C), the interfacial force grows sharply. Such rapid growth of the interfacial force is the result of another positive feedback loop involving curvature-enhanced PIP_2_ hydrolysis. We schematize the qualitative mechanism in Figure 4B. As the initial interfacial force squeezes the neck, it creates a higher mean curvature at the interface. The higher the mean curvature of the membrane, the more PIP_2_ is exposed and susceptible to phosphatase activity. Consequently, more PIP_2_ is depleted at the interface region along the membrane invagination. Thus, a larger difference in local PIP_2_ levels bounding this location is induced (∼21–27 s, Figure 5B and 5C), which in turn speeds up the growth of the interfacial force and, hence, further squeezes the interface. This is a self-accelerating process.

The sharp dip of the PIP_2_ levels around the bud-tubule interface compared to the smaller difference between those of tubule and bud (t∼23 s and 27 s in Figure 5C) suggests that curvature-dependent PIP_2_ hydrolysis is the predominant driving force for generating the interfacial force. Our model thus predicts that the pinching force arises as a result of differential phosphatase activity along the membrane invagination. This prediction is consistent with the observations that phosphatase activity is essential for endocytic vesicle scission in yeast, that the phosphatase concentrates at the endocytic site during the late stages of endocytic vesicle internalization, and that it moves into the cell with the forming vesicle, possibly suggesting enrichment at the vesicle tip [11].

During t∼27–29 s (Figure 5B and 5C), as the pinching force squeezes the neck, the membrane curvature in the radial direction of the tubule deviates from the optimal shape for BDP binding (t∼28 s and 29 s in Figure 3B). This deviation acts as a “disassembly signal” and invokes the intrinsic positive feedback loop between curvature sensing and curvature deforming of BDPs (Figure 4A), triggering the rapid BDPs turnover (∼27–29 s in Figure 3A). Meanwhile, PIP_2_ gets hydrolyzed not only at the bud but also on the tubule due to the lack of BDP protection (t∼29 s, Figure 5C). Although this leads to a fast decrease in the interfacial force (∼27–29 s, Figure 5B), the pinching force is still sufficient to drive rapid vesicle scission according to our calculations.

We need to point out that, while the in vitro systems on lipid phase segregation are crucial for identifying mechanical forces that might be involved in vesicle scission, the experimental conditions used are quite different from the in vivo conditions during endocytosis. Once the lipid phase segregation takes place in the in vitro systems, the resulting interfacial force persists and there is no time limit for the vesicle scission process. All that matters is that the interfacial force needs to be sufficiently large to overcome the membrane bending resistance [24],[50]. In cells, the timing of the lipid phase segregation is predicted to be critical for successful endocytosis. The threshold interfacial force value required for scission can be determined by force-balance calculations [24],[50]. A rapid nonlinear time course for interfacial force development in endocytosis means that successful scission in vivo can only occur within a short time window (the shaded region in Figure 5B).

### Successful Endocytosis Depends on the Feedback between Local Chemical Reactions and Membrane Shape Changes

**Figure 6:**
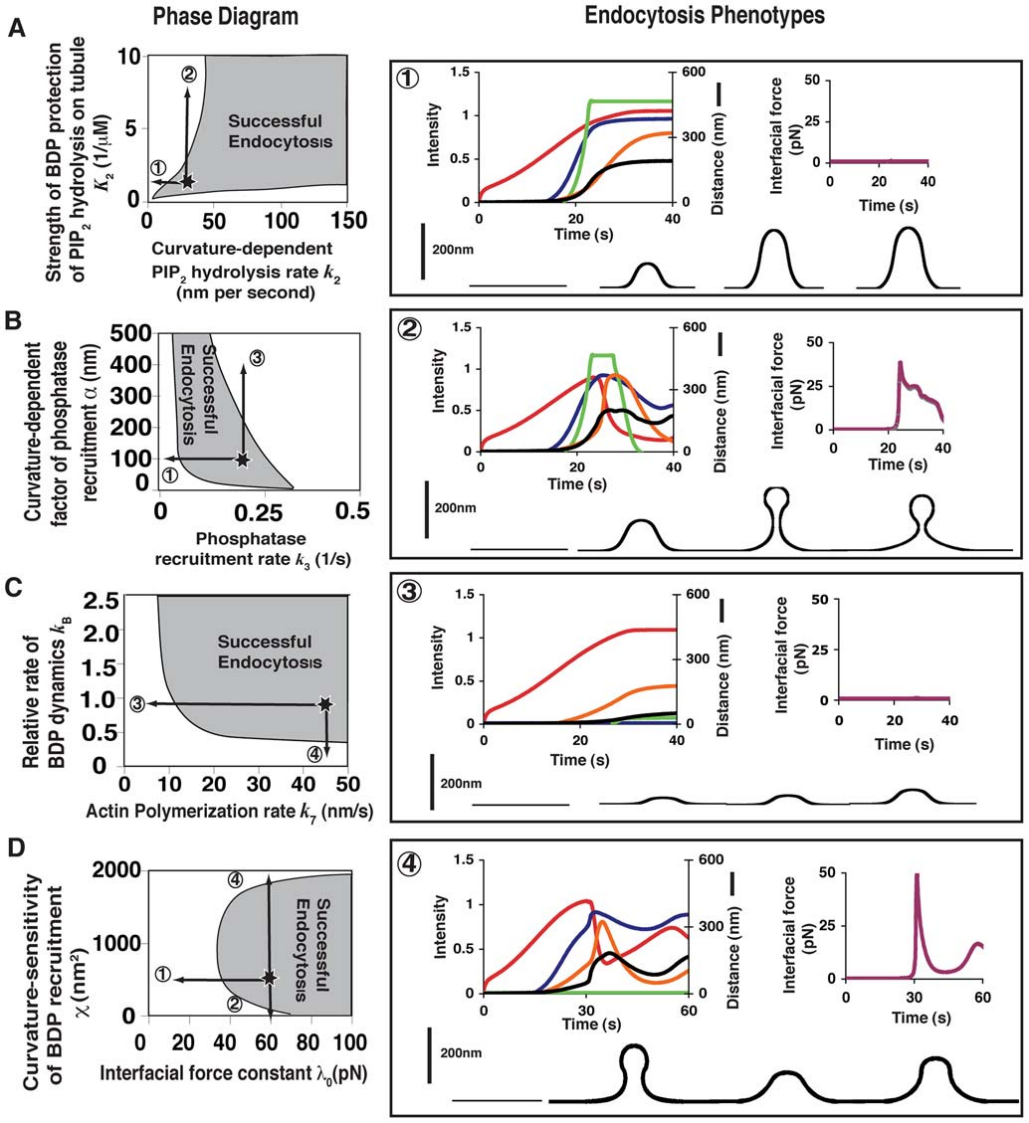
Phase diagrams for endocytic dynamics. The shaded areas represent the parameter regions for successful endocytosis; the star in each phase diagram represents the parameter set used in the fitting plot in Figure 3. (A) Strength of BDP PIP_2_ protection: *K*_2_ versus curvature-dependent PIP_2_ hydrolysis rate *k*_2_; (B) Curvature-dependent factor for phosphatase recruitment rate, α versus phosphatase recruitment rate *k*_3_; (C) Relative rate of BDP dynamics versus actin polymerization rate *k*_7_; (D) Curvature-dependent factor of BDP recruitment rate χ versus interfacial force constant *λ*_0_. Each phenotype is characterized by: (a) time-lapse plot for the coat proteins (red), actin (blue), BDP (green), phosphatase (orange), and the membrane tip position (black); (b) the time course for interfacial force development (purple); (c) the time course for membrane shape change (black). The intensity of each functional module in the phenotype plots is normalized relative to the corresponding wild-type normalized intensity shown in Figure 3, thus representing the relative abundance. Phenotype 1: Without PIP_2_ hydrolysis [*k_2_* reduces from 20 (nm) per second to 0]. Phenotype 2: Increased protection strength of PIP_2_ hydrolysis at the tubule region [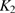 increases from 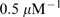 to 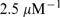]. Phenotype 3: Increased phosphatase recruitment rate [*α* increases from 100 nm to 500 nm]. Phenotype 4: BDP recruitment does not occur.

In this section, we will explore in detail how mechanochemical feedback ensures the precise timing and sequence of endocytic events and guarantees rapid endocytic vesicle scission. In Figure 6A–6D phase diagrams for endocytosis are computed for different pairs of model parameters. These diagrams serve several purposes. First, they show that the model is robust: it can generate successful endocytosis over a large range of the parameters. Second, equipped with these phase diagrams, we can vary the parameters to mimic the conditions of mutant experiments. Third, they constitute an independent experimental test of the model. This is because the identities of the functional modules were in part derived from mutant experiments, but we did not explicitly take into account the mutant phenotypes in the model. That is, we used the five time-lapse curves and membrane shape changes to determine the four free parameters in the model, and then used these parameter values to predict mutant phenotypes. Thus, these predictions are independent of the parameter set, and consequently the agreement between predicted and observed phenotypes constitutes cross-validation of the model. Finally, based on the calculated phase diagrams, we can predict endocytic phenotypes for mutants that have not yet been made, thus guiding further experiments.

### Dependence on Fast PIP_2_ Hydrolysis Rate on Membrane Curvature

Figure 6A shows that endocytosis can only be successful when the curvature-dependent PIP_2_ hydrolysis rate is sufficiently fast. Otherwise, the PIP_2_ level difference across the interfacial boundary will not have had sufficient time to grow before the membrane bending energy resists squeezing and quickly balances the interfacial force without triggering the positive feedback loop (Figure 4B). Accordingly, the absence of positive feedback between the interfacial force and the local membrane curvature leads to a distinct phenotype (*phenotype 1*, wherein the PIP_2_ hydrolysis rate *k*_2_ is reduced from 20 (nm) per second to zero): F-actin associated forces could still drive membrane invagination; the interfacial force, however, would not squeeze the neck effectively, because the force cannot grow large enough. Thus, the whole system would eventually reach a mechanochemical equilibrium wherein a slightly curved membrane invagination could persist for a time without vesicle scission. This phenotype is consistent with the budding yeast mutant *sjl1Δ sjl2Δ* [11], wherein the PIP_2_ hydrolysis is dramatically reduced.

If the PIP_2_ hydrolysis rate is very fast but independent of the local membrane curvature, then the positive feedback between the interfacial force and the local membrane curvature is ablated (see Figure 4B and 45). Without this positive feedback, the interfacial force would always remain at its initial basal level, which is insufficient to pinch off the vesicle (see details in Figure S2). Successful endocytosis, therefore, requires the positive feedback between interfacial force and curvature-dependent PIP_2_ hydrolysis activity. This is further dictated by two conditions: first, the PIP_2_ hydrolysis rate must be faster than the typical response time scale of the membrane, and second, PIP_2_ hydrolysis must be curvature-dependent. The former can be tuned by the local concentration of phosphatases, and the latter is intrinsic to the mechanism of enzyme activity.

### Proper Protection of Tubule PIP_2_ by BDPs Is Essential for Endocytosis

Figure 6A shows that, even with a sufficiently high curvature-dependent PIP_2_ hydrolysis rate, endocytosis may not be successful unless the protection of PIP_2_ at the tubule by BDPs is sufficiently effective (large *K*_2_). Otherwise (small *K*_2_), the resulting interfacial force would be too small to drive vesicle scission. On the other hand, if the protection is too effective, then PIP_2_ levels at the tubule would be maintained at a high level, which in turn would lead to persistent BDP accumulation. As BDPs tend to deform the membrane to a specific, preferred shape (diameter ∼30 nm), persistence of the BDPs would effectively hold the neck and prevent any further narrowing of the membrane tubule, hindering vesicle scission. This leads to prediction of a unique phenotype (*phenotype 2*, wherein the protection strength of PIP_2_ hydrolysis at the tubule region 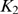 increases from 0.5 μM^−1^ to 2.5 μM^−1^), in which the absolute levels and the lifetimes of the BDPs would increase significantly as compared with the wild-type situation. Furthermore, a long and narrow membrane invagination could persist without vesicle scission. This is because BDPs have their own preferred shape (a tubule of ∼30 nm in diameter), and their persistence would tend to preserve the shape of membrane tubule, preventing any further squeezing in response to the interfacial force.

### Dependence on the Timing of Phosphatase Recruitment

Our model predicts that within the successful endocytosis region in Figure 6A, increasing the curvature-dependent PIP_2_ hydrolysis rate *k*_2_ will speed up endocytosis and that this effect will saturate at large *k*_2_. This is because in this case endocytic dynamics are limited by the phosphatase recruitment rate, instead of by its activity. As shown in Figure 6B, positive feedback between interfacial force development and local membrane curvature will not develop if the phosphatase activity is not sufficient. Insufficient phosphatase results in a phenotype similar to those observed when PIP_2_ hydrolysis curvature dependence is insufficient, as shown in Figure 6A, and/or when PIP_2_ hydrolysis is independent of curvature, as shown in Figure S2.

On the other hand, endocytosis will also be impeded if the phosphatase is overexpressed or overactive at the endocytic site, which leads to *phenotype 3* (where the curvature-dependent factor of phosphatase recruitment rate *α* increases from 100 nm to 500 nm). Here scission fails because the excessive phosphatase diminishes the initial PIP_2_ level difference across the bud-tubule boundary, thus preventing the development of the initial squeezing force. As a result, the membrane at the interface cannot be deformed sufficiently to invoke positive feedback between interfacial force development and the curvature-dependent PIP_2_ hydrolysis activity.

A surprising conclusion from our model is that coat proteins will still assemble at the endocytic site in the presence of excessive phosphatase and will disassemble slowly. This conclusion is based on the linear dependence of the PIP_2_ hydrolysis rate on the local membrane curvature, which is in accordance to experimental observations. PIP_2_ hydrolysis is relatively slow despite high phosphatase levels because the membrane is not highly curved (e.g., phenotype 3). Thus, even though the phosphatase recruitment is very fast in phenotype 3, its action is limited by the lack of membrane curvature, which is low because a pronounced phase boundary does not develop.

### Endocytosis Critically Depends on Coordination between BDP Recruitment and F-Actin Polymerization

Figure 6C shows that successful endocytosis also critically depends on the coordinated dynamics of BDP recruitment and F-actin polymerization. Without actin polymerization, the endocytic membrane cannot become deeply invaginated. Failure to invaginate the membrane prevents BDP accumulation and the ensuing development of the interfacial force. Consequently, the membrane cannot deform into a deep invagination, nor proceed to vesicle scission. This situation is similar to having excessive phosphatase at the endocytic site, leading to phenotype 3 in Figure 6, consistent with actin-assembly inhibition phenotype in budding yeast [8].

When actin polymerizes normally, efficient endocytosis requires sufficiently fast BDP accumulation. Insufficient BDP recruitment would lead to *phenotype 4* (wherein the BDP recruitment rate drops to zero): the endocytic membrane will be pulled out and will then retract without vesicle scission (a movie of the process is given in Video S2). This is because although the peak interfacial force is large enough to squeeze the neck in phenotype 4, the force declines so rapidly that the membrane does not have time to undergo deformation and, hence, the vesicle cannot be successfully pinched off. A large interfacial force can develop in the absence of the BDPs in phenotype 4 because the actin filaments contact actin-binding proteins associated with the coat so that the actin pulling force impinges on the entire bud region of the endocytic membrane, including the bud-tubule boundary. Although very small, the force from the actin module can still deform the membrane at the neck slightly, which activates the curvature-dependent PIP_2_ phosphatase activity. Hence, the positive feedback loop is triggered, leading to generation of a large interfacial force. However, without BDP protection, this large interfacial force is too short-lived and vesicle scission does not occur.

On the other hand, in the absence of sufficient numbers of BDPs, the high curvature of the membrane invagination generated by F-actin polymerization would still induce phosphatase recruitment, which would result in disassembly of the entire endocytic apparatus and retraction of the membrane invagination. This predicted phenotype is consistent with the phenotype of a budding yeast *rvs167* (a BDP) knockout mutant [9] and a lipid-binding defective *rvs167* point mutant (Kishimoto and Drubin, unpublished).

### Interplay between the Interfacial Force and BDP Turnover

The lifetime of BDPs at endocytic sites is extremely short (∼10 s) in wild-type budding yeast [9],[11]. We have shown for phenotype 2 of Figure 6A that prolonged accumulation of BDPs could prevent endocytosis. A key message emerging from these two observations is that the interplay between the interfacial force and BDP turnover is critical for successful endocytosis. As the interfacial force squeezes the interface, it tends to narrow the adjacent membrane tubule, which deviates from the shape preferred by BDPs. This deviation leads to a curvature mismatch and acts as a “disassembly” signal for the BDPs as dictated by the BDP sensitivity factor (the exponential term χ in Equation 5). Accordingly, upon narrowing of the tubule, the higher the sensitivity factor χ, the faster the turnover of the BDPs, and hence the more that vesicle scission is facilitated. As Figure 6D shows, when the interfacial force is very large (>60 pN), it is capable of squeezing the interfacial boundary even if the BDPs are not disassembled; endocytosis would proceed normally even with prolonged BDP accumulation at the tubule (χ = 0). On the other hand, when the interfacial force is in an intermediate range (e.g., 30–60 pN), its action could be insufficient to overcome the bending resistance of the preferred membrane shape set by the BDPs. Given that the interfacial force will also dissipate in a short period of time (∼5 s, Figure 5A), a minimal level of curvature-dependent sensitivity in BDP accumulation is required to induce fast BDP turnover upon squeezing of the membrane tubule, relieving the bending resistance, and hence facilitating vesicle scission. This sets the lower threshold of the curvature-dependent sensitivity of BDP dynamics for successful vesicle scission. Note that the curvature sensitivity, χ, is central to the positive feedback between BDP recruitment and the local membrane deformation (Figure 4A). The above results imply that successful endocytosis requires that BDP binding feeds back positively with the underlying membrane shape.

## Discussion

### Mechanochemical Feedback Is Critical for Ensuring Successful Endocytosis in Budding Yeast

During endocytosis, recruitment of the endocytic proteins is sequential and self-reinforcing, or autocatalytic [3],[4],[8],[9],[10],[11],[12],[35]. We propose that these features are properties of positive mechanochemical feedback loops between membrane curvature and the various reactions leading to vesicle formation and scission (Figures 2–6). To our knowledge, our model is the first of its kind that can coherently capture all of the key endocytic events in budding yeast. The dynamics predicted by the model fit well with time-lapse experimental measurements (Figure 1A). Moreover, the parameter diagrams in Figure 6 show that successful endocytosis can be realized over a broad range of parameter space. Thus the endocytic process is largely buffered against variations in the activities of specific molecular players.

**Figure 7:**
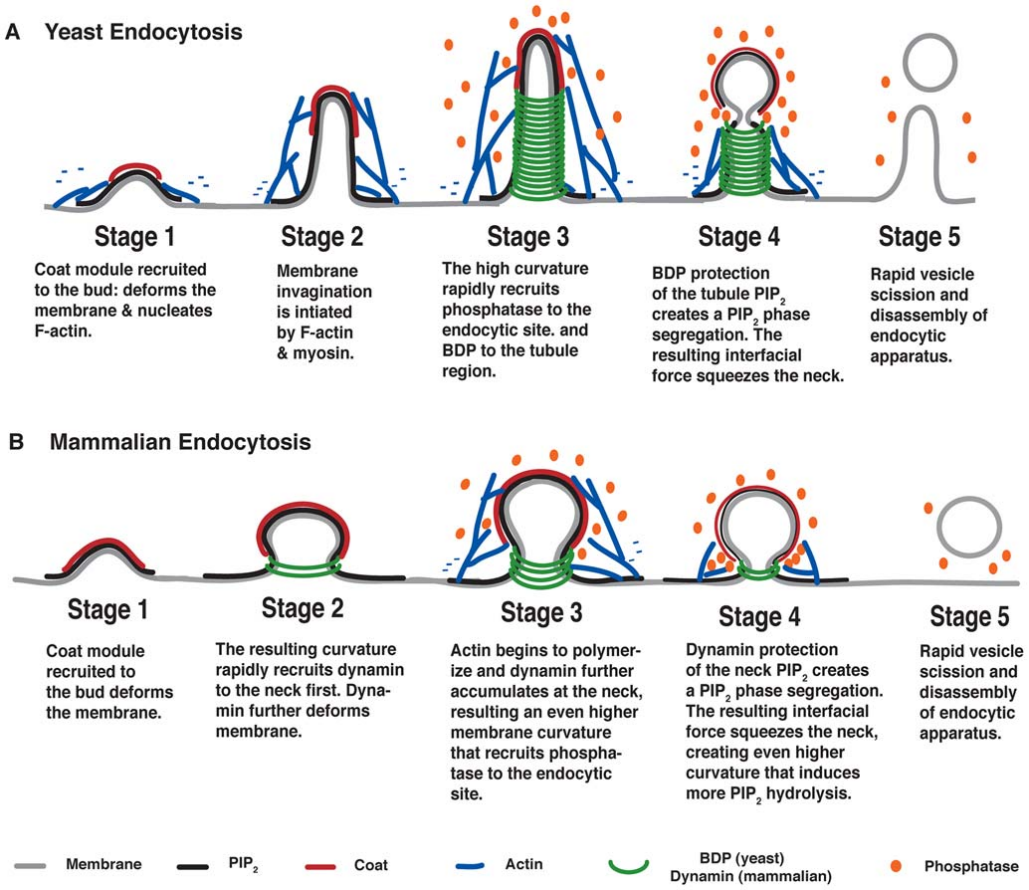
Schematics comparing endocytosis in yeast and mammalian cells. (A) Model for yeast endocytosis. (B) Model for mammalian endocytosis (see text).

Endocytosis in budding yeast evolves in a sequence of events that are explained by the model (as schematized in Figure 7A). As PIP_2_ accumulates at the endocytic site, it recruits coat proteins to the bud region that nucleate actin polymerization. Using anchorage to the coat proteins (e.g., Sla2), F-actin polymerization and myosin motor activity generate a pulling force that deforms the membrane into a tubule. The high curvature of the tubule in turn recruits BDPs that coat the tubule by binding to PIP_2_. The BDPs protect the PIP_2_ along the tubule from hydrolysis by the phosphatase. The coat proteins on the vesicle bud do not protect the PIP_2_ from hydrolysis as effectively, so a boundary region is created that develops a circumferential interfacial tension. This tension exerts a squeezing force on the phase boundary, which further increases the curvature at the bud neck, which in turn increases the hydrolysis there. Thus a positive feedback loop arises between membrane curvature and PIP_2_ hydrolysis rates at the interface, the result of which is the rapid growth of the interfacial force leading to vesicle scission (Figure 5). Furthermore, the positive feedback loop between the curvature-sensing and deforming activities of the BDPs ensures rapid turnover of the BDPs, facilitating timely vesicle scission. After scission, PIP_2_ is hydrolyzed all over the membrane surface, promoting disassembly of the entire endocytic apparatus. Therefore, it is the two intertwined positive feedback loops (Figure 4) that ensure rapid, robust, and timely endocytosis in budding yeast.

### Clathrin-Mediated Endocytosis in Mammalian Cells

Our model depicts endocytosis at the level of functional modules, rather than at the level of particular proteins; the model enables us to discern the general features of the process and to dissect how the sub-processes fit together. As different proteins can play the same functional role in different organisms, our model can be extended to account for the endocytosis in other organisms. We have applied this framework to endocytosis in mammalian cells; the predictions from our model are largely consistent with experiments and provide further mechanistic insight, suggesting that similar principles may dictate the dynamics and robustness of protein recruitment, and the vesicle scission mechanism.

Our model predicts that the main profile of the endocytic membrane in mammalian cells is a constricted coated pit instead of the tubular structure in yeast. The interfacial force generated by lipid phase segregation is sufficient to pinch off the vesicle, and actin is largely dispensable while the membrane-deforming dynamin GTPase and clathrin are essential. We schematize our main findings of mammalian endocytosis in Figure 7B and relegate the detailed discussions to Protocol S1.

### Predictions of the Model

The model reproduces the behavior of observed endocytic mutant phenotypes and predicts several phenotypes that have not yet been studied in experiments. We predict that yeast endocytosis will be hindered if BDP protection of PIP_2_ on the tubule is either too weak or too strong, which is testable by BDP mutant analysis. Weak protection of PIP_2_ would reduce the PIP_2_ difference and, hence, the interfacial squeezing force. On the other hand, the more persistently the BDPs coat the tubule, the more resistant the tubule will be to the further squeezing from the interfacial force (phenotype 2 in Figure 6). This is because BDPs prefer a well-defined membrane shape (tubules of 30 nm diameter). In addition to rapid BDP assembly, therefore, BDP disassembly concurrent with vesicle scission is also essential for endocytosis.

The role of BDPs in vesicle scission suggests an explanation for dynamin mechanism that contrasts with the conventional view of dynamin as a pinchase (see Section F in Protocol S1 for a detailed discussion of dynamin). Dynamin disassembly precedes membrane fission [51],[52], which suggests that dynamin may act to disrupt local membrane structure, perhaps through generation of a phase boundary. Disassembly would be required to release the underlying membrane, allowing a line tension to constrict the vesicle neck to drive scission.

Successful endocytosis also entails three constraints on PIP_2_ hydrolysis rates, all of which lie at the heart of the mechanochemical feedback loop and can be tested by in vivo and in vitro experiments. First, the PIP_2_ hydrolysis rate must be curvature-dependent (see Figure S2). Second, it must be faster than the response time scale of the membrane deformation (Figure 6A). Third, it must be slower than the time scale for assembling the endocytic apparatus (Figure 6B). We predict that when the PIP_2_ hydrolysis rate drops below a threshold, endocytosis will cease, but the endocytic membrane invagination will persist (phenotype 1 in Figure 6). Thus, the phosphatase not only uncoats proteins from the endocytic vesicle, but it also is essential for vesicle scission. This dual function makes sense because endocytosis is a sequential process: each step paves the way for the next one. The coat proteins on the bud must disassemble upon—or shortly after—vesicle scission. Uncoating is essential for the fusion of endocytic vesicle with early endosomes and coat protein recycling. This prediction provides a fresh perspective on the functions of phosphatase/lipase in endocytosis in yeast as well as in mammalian cells, e.g., synaptojanin in neurons [36].

### Curvature Control Makes the Endocytic Process Robust

Given the small number of proteins present at each endocytic site at different times in the process (∼10–100) [10],[53], it would appear that the process should be very stochastic. Typically, stochastic protein recruitment arises from variations in the assembly “source signal” and in the number of proteins being recruited. The rapid sequential recruitment of endocytic proteins, such as the BDPs and phosphatase, implies a highly cooperative process: the Hill coefficient for BDP recruitment by actin is >6 as inferred from [9],[10]. Thus, without compensating mechanisms, small variations in the source signal would be amplified to large uncertainties in recruitment. And yet the timing of endocytic protein recruitment is very robust, and endocytosis proceeds smoothly. The effects of small variations in protein levels and activity could be overcome if extremely specific protein-protein interactions acted as a template for recruitment, which requires the free energy decrease for protein binding to be well above the level of thermal fluctuations, i.e., >10 k_B_T.

Our model implies an alternative mechanism: using local membrane curvature as the source signal; i.e., to assemble and disassemble BDPs. If we add random noise to Equations 1–5 and Equation 6 to mimic the instantaneous fluctuations in protein numbers and membrane shape fluctuations, respectively, endocytosis remains stable up to 20%–30% variation in the maximum levels for each module (unpublished data). The reason for this stability is the small diameter of the endocytic invagination (∼50 nm). On this scale, the membrane is quite stiff, and so the membrane curvature will not fluctuate much because of the energy penalty associated with stochastic fluctuations in membrane shape (∼100 k_B_T) [54],[55]. Moreover, since a curvature mismatch increases the free energy associated with BDP binding, the membrane curvature modulates the BDP recruitment rate via a Boltzmann factor (Equation 5). Thus, the local membrane curvature is instantaneously stable throughout the process and dictates the timing and location of BDP assembly and disassembly accurately despite stochastic fluctuations. Hence, the mechanochemical feedback has a build-in robustness that ensures successful endocytosis.

### Future Directions

In the future, much experimental and theoretical work will be required to test and refine our model. Here we discuss aspects of our model for budding yeast endocytosis that we have not yet addressed. A related discussion for mammalian cells is presented in Section F in Protocol S1.

For our model, the key to promoting rapid vesicle scission was to invoke positive feedback between growth of the interfacial force and curvature-dependent PIP_2_ hydrolysis at the interfacial boundary, resulting in a sharp dip in the local PIP_2_ concentration at the interface. For this mechanism, all that is needed is to induce a localized membrane deformation (i.e., higher mean curvature) at a specific site along the membrane tubule. This in turn will trigger a positive feedback effect on PIP_2_ hydrolysis. There are many ways in which a localized membrane deformation can be generated. In this paper, we only entertained one scenario, in which the initial squeezing of the membrane at the interfacial boundary is the result of an initial PIP_2_ level difference (lower in the bud region) due to BDP protection of PIP_2_ hydrolysis on the tubule. However, other scenarios are also feasible. For instance, as phenotype 4 shows, even without BDPs, the impact from normal actin/myosin force could deform the membrane neck so as to invoke positive feedback and hence a large interfacial force. Although in this case the interfacial force is too short-lived to drive vesicle scission, this scenario nonetheless suggests other avenues to generate a sufficiently strong and persistent force. Also, it could be that the coat proteins protect PIP_2_ on the bud more effectively than the BDPs protect PIP_2_ on the tubule. This will result in a higher PIP_2_ level at the bud relative to the tubule, which could equally well induce an interfacial force. Although this scenario seems less likely due to the apparent concentration of the phosphatase at the bud tip, clearly experimental work is needed to determine how yeast pinch off endocytic vesicles in the absence of dynamin.

Also, studies on the mechanisms that recruit PIP_2_ phosphatases to endocytic sites are needed. In fact, actin has been shown to recruit the phosphatase via the actin-binding protein Abp1 [11],[35], although this effect alone cannot account for the full phosphatase recruitment to the endocytic site in yeast [11]. What is not clear is whether actin or the actin-dependent membrane curvature, or the combined effects, are responsible for PIP_2_ phosphatase recruitment. In our model, we treated PIP_2_ phosphatase recruitment as curvature dependent without delving into the specific contributions of direct actin-mediated recruitment versus indirect membrane curvature-dependent recruitment. We can show that the curvature-dependence of PIP_2_ phosphatase recruitment is not essential for efficient endocytosis as long as the effective phosphatase recruitment rate is neither too fast nor too slow as compared to PIP_2_ synthesis (Figure S4) and the hydrolysis rate is curvature-dependent. Future experimental studies must mechanistically address the contributions of BDPs, actin, and coat proteins in the vesicle formation process.

In summary, our model is based on the notion that the local curvature of the endocytic membrane is both slave to, and master over, the accompanying biochemical reaction pathways. The coupling between curvature and biochemical reactions orchestrates a robust sequence of events leading to vesicle scission. Formulating the model in terms of functional modules allowed us to look beyond the molecular details and explore the larger features of how membrane dynamics and biochemical reactions fit together during endocytosis. This scheme can quantitatively describe clathrin-mediated endocytosis in budding yeast and the analogous process in mammalian cells. Thus, our model can serve as a unified framework for dissecting endocytosis in general.

## Materials and Methods

### Mathematical Description of the Model

We incorporate the qualitative ingredients of the model into a set of quantitative equations. The detailed assumptions and the choices of the parameters are given in Protocol S1. Equations 1–5 describe the dynamics of the chemical reactions of the functional modules on the surface of the endocytic membrane. Levels of functional modules are expressed as the coverage fraction (mole fraction). We assume that the endocytic membrane has cylindrical symmetry. The local spatial coordinates along the membrane surface represent the arc length *s* with unit length 1 nm. The local membrane shape is uniquely defined by the tangent angle *ϕ*(*s*) and the radius *r*(*s*) (see Figure 2). The bud region is defined by the arc length *s* = 0–100; the tubule region is defined by *s* = 101–500.

PIP_2_ dynamics in the bud region (Notation: ***P***):
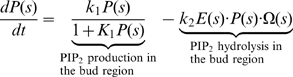
(1a)


PIP_2_ dynamics in the tubule region:
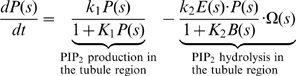
(1b)


Enzyme (lipid phosphatase or lipase) dynamics (Notation: ***E***):
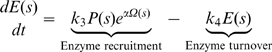
(2)


Coat protein dynamics in the bud region (Notation: ***C***):
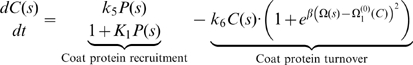
(3)


Actin dynamics in the bud region (Notation: ***A***):
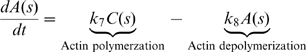
(4)


BDP dynamics in the tubule region (Notation: ***B***):
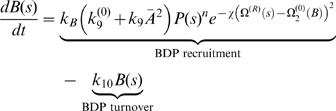
(5)


In Equations 1–5, Ω(*s*) and Ω^(*R*)^(*s*) are the mean curvature and the curvature in radial direction of the local membrane invagination, respectively, which are defined by local membrane orientation *ϕ*(*s*) and radius *r*(*s*) (see Figure 2 and Protocol S1 for their formula). 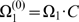 and 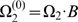 are the preferred curvatures by coat proteins at the bud and by the BDPs at the tubule, respectively. Ω*_C_* and Ω*_B_* are the preferred curvatures for the bud region and the tubule region, respectively, when they are fully covered by their corresponding proteins (*C* = 1, *B* = 1). The key mechanochemical couplings are: the PIP_2_ hydrolysis rate linearly depends on the local membrane curvature in Equation 1; BDP recruitment rate depends exponentially on its fit to the local membrane curvature in Equation 5. Furthermore, 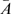 term in Equation 5 represents the actin-aided BDP recruitment, where 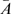 is the average actin level at the endocytic site (see Protocol S1 for details).

The feedback between the chemical reactions and the membrane shape is specified by how the local chemical reactions directly control the membrane dynamics. The membrane dynamics is governed by Equation 6:
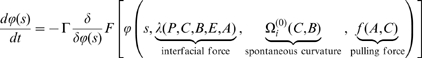
(6)Here, *F*[*ϕ* (*s*)] is the Helfrich-like free energy for the endocytic membrane, which is characterized by the membrane bending energy and surface tension that specify the energy penalty associated with membrane deformations. Γ is the relative timescale of the membrane dynamics compared to the local chemical reactions.

Equation 6 describes the membrane dynamics affected by the interfacial force *λ*, the spontaneous curvatures 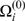, and the pulling force *f* in the bud region, which are all controlled by the local chemical reactions. The interfacial force *λ* is a function of the PIP_2_ level difference across the interface between the bud region and the tubule region, 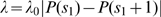, where *λ*_0_ is the interfacial force constant and *s* = 100 is the interfacial boundary position (see Figure 2). Note that the pulling force on the bud region must anchor to the coat protein to be effective. We neglect protein diffusion in Equations 1–5 and the in-plane hydrodynamics of membrane flow in Equation 6. The justifications for these assumptions are given in Protocol S1.

## Supporting Information

Protocol S1 **Supplemental data.** This file includes six sections. (A) Details of theoretical model assumption and derivations. (B) Table of model parameters. (C) Membrane free energy functional. (D) Membrane tubulation driven by BDPs binding. (E) Additional phase diagrams. (F) The functional module description can account for endocytosis in mammalian cells.
(0.58 MB DOC)

Figure S1 **Calculated membrane tubulation driven by BDPs binding.** The calculation is carried out in 3-D, and the membrane profile is shown in 2-D. The simulation shows four stages in the growth of the tubule (labeled by time step 1–4). The initial condition is a flat membrane patch, an infinitely large reservoir of BDPs, and there are no membrane-bound BDPs (please see Section D in Protocol S1 for details).
(0.23 MB TIF)

Figure S2
**Phase diagram for the fate of endocytosis in budding yeast characterized by the curvature-dependent and the curvature-independent PIP_2_ hydrolysis rates.** Note that since the curvature involved in the membrane invagination is typically ∼1/(100 nm), the range of the curvature-dependent PIP_2_ hydrolysis rate in Figure S2 is ∼0–0.3 per second per phosphatase that is comparable with 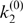 (please see Section E(I) in Protocol S1 for details).(0.15 MB TIF)

Figure S3 **Phase diagram for endocytosis in budding yeast characterized by the relative timescale of membrane dynamics and the interfacial force constant (please see Section E(II) in Protocol S1 for details).**
(0.10 MB TIF)

Figure S4 **Phase diagram for the fate of endocytosis in budding yeast characterized by PIP_2_ synthesis rate and PIP_2_ phosphatase turnover rate (please see Section E(III) in Protocol S1 for details).**
(0.10 MB TIF)

Figure S5 **The interaction diagram amongst the functional modules in mammalian endocytosis (please see Section F in Protocol S1 for details).**
(0.45 MB TIF)

Figure S6 **Endocytosis dynamics for mammalian cells.** (A) Calculated time-lapse of the functional modules and the tip position of the endocytic membrane. (B) Snapshots of the calculated endocytic membrane shape changes. (C) The development of interfacial force over time. In contrast to budding yeast, the recruitment rate of dynamin is independent of actin and is much faster: we take it to be 4.0/s; actin polymerization and depolymerization rates are slowed down by 2-folds (22.5 nm/s and 15 nm/s, respectively). If not otherwise specified, the other parameters in this modified model are the same as those for yeast endocytosis (please see Section F in Protocol S1 for details).
(0.37 MB TIF)

Figure S7 **Phase diagram for mammalian endocytosis characterized by actin polymerization rate and the recruitment rate of dynamin.** Note that there is a threshold value of dynamin recruitment rate ∼0.1/s, only above which endocytosis can be successful. Due to the resolution of the scales in *y*-axis, it is not shown here (please see Section F in Protocol S1 for details).
(0.11 MB TIF)

Figure S8 **Predicted clathrin knock phenotype in mammalian endocytosis.** (A) Calculated time-lapse of the functional modules and the tip position of the endocytic membrane. (B) Snapshots of the calculated endocytic membrane shape changes. (C) The development of interfacial force over time. Here the recruitment rate of the coat protein at the bud is taken to be zero. The intensities of the proteins in (A) are normalized relative to those in Figure S4 (please see Section F in Protocol S1 for details).
(0.25 MB TIF)

Figure S9 **Predicted dynamin knockout phenotype in mammalian endocytosis.** (A) Calculated time-lapse of the functional modules and the tip position of the endocytic membrane. (B) Snapshots of the calculated endocytic membrane shape changes. (C) The development of interfacial force over time. Here the dynamin recruitment rate is taken to be zero (please see Section F in Protocol S1 for details).
(0.28 MB TIF)

Video S1 **Computed wild-type endocytosis in budding yeast.** The movie shows the calculated endocytic membrane shape change in wild-type yeast during the same time course as that depicted in the curve fitting plots of Figure 3 in the main text. The parameters used in the calculations are listed in Table 1 of Section B in Protocol S1; the initial endocytic membrane profile is flat. The unit for both the *x*- and *y*-axes in the movie is nm, and 1 s in the movie corresponds to 2.5 s in real time.
(0.08 MB MOV)

Video S2 **Computed yo-yo phenotype of endocytosis in budding yeast.** The movie shows the calculated endocytic membrane shape change during the same time course of that in phenotype 4 of Figure 6 in the main text. The parameters used in the calculation are listed in Table 1 of Section B in Protocol S1, except that BDP recruitment does not occur. The initial endocytic membrane profile is flat. The unit for both the *x*- and *y*-axes in the movie is nm, and 1 s in the movie corresponds to 4.5 s in real time.
(0.06 MB MOV)

## Abbreviations

BAR: Bin/Amphiphysin/Rvs
BDP: BAR Domain Protein
