# Supplementary material for: The Mechanochemistry of Endocytosis
Source: PLoS Biol. 2009 Sep 29;7(9):e1000204. doi: 10.1371/journal.pbio.1000204 (PMC2742711; doi:10.1371/journal.pbio.1000204)
Supplement: Protocol S1 — Supplemental data. This file includes six sections. (A) Details of theoretical model assumption and derivations. (B) Table of model parameters. (C) Membrane free energy functional. (D) Membrane tubulation driven by BDPs binding. (E) Additional phase diagrams. (F) The functional module description can account for endocytosis in mammalian cells. (0.58 MB DOC) [file pbio.1000204.s001.doc]

# A. Assumptions and derivations

Our model is based on the notion of *functional modules*. In principle, each functional role could be played by any of several proteins or lipids. The kinetic parameters that characterize the dynamics of each functional module represent the overall effect of different processes within that module. The local levels of the proteins are scaled to the fractional coverage per unit area of the membrane surface. The model assumes rotational symmetry of the tubule and bud, the units are (arc length)^2^, where the unit arc length is 1 nm. Some of the measured kinetic parameters are converted into the unified scale adopted by the model. All the enzymatic kinetic steps in the model equations are treated as Michaelis-Menten reactions. The conversion procedure is as follows.

**1**. We model the local PIP_2_ synthesis as follows. Adaptor proteins bind to PIP_2_ and rapidly reach equilibrium with affinity 1/*K*_1_, where *K*­_1_ is the Michaelis-Menten constant. The bound adaptor protein then recruits phosphoinositol kinase (e.g. PIP5 kinase) that converts PIP into PIP_2_ with apparent rate *k*_1_ [1]. Hence, the local phosphoinositol kinase level is proportional to in Eq. [1]. From Michaelis-Menten kinetics, the PIP5 kinase activity is , where $c_{S}$ is the concentration of the substrate, i.e. PI4P in our case, and , where $c_{E}$ is the concentration of the enzyme, i.e. PIP5 kinase. From the measured $V_{\text{max}}$[2,3,4], we can obtain the PIP5 kinase activity as: 0.0004-0.007/s. There are two effects we need to consider. The first is the size of PIP5 kinase ~70-100kDa [2]. When synthesizing PIP_2_, the kinase typically covers ~ 100 lipids while binding to the membrane. This will introduce ~100 fold activity increase in the scale used in the model. The second effect is the ~ 10 fold stimulation factor induced by phosphatidic acids (PA) [2], which are abundant on the plasma membrane. Combining the two effects, PIP_2_ synthesis rate is $k_{1}$ ~ (0.4-7)/s.

**2**. The PIP_2_ hydrolysis rate (*V*_max_) by inositol phosphatase (e.g. synaptojanin) and lipase (e.g. phospholipase C) ranges from 125 nmol to 12 μmol per min per mg [5,6,7,8,9]. Given the molecular weight of synaptojanin ~ 115 kDa, and that of phospholipase C ~80 kDa [5,6,7,8,9], the PIP_2_ hydrolysis rate is converted to 0.3 ~ 30 PIP_2_/s. These values are measured *in vitro*, where the enzymes hydrolyze pure PIP_2_ in solution. Under such conditions, PIP_2_ typically forms micelles of ~ 20 nm diameter [10]. *In vitro* experiments show that phosphoinositide hydrolysis rate depends linearly on the mean curvature of the membrane Ω [11]. The physical interpretation is: As the membrane curvature increases, the phosphoinositide lipids splay more, and hence increases access of the lipase. Thus, membrane curvature speeds up the activity of the lipase. We further propose that a similar mechanism operates for PIP_2_ hydrolysis; this has been corroborated experimentally by [Chang et al., *personal communication*]. In the model, the apparent PIP_2_ hydrolysis rate *k*_2_ is chosen such that$k_{2}\Omega$ lies in the range 0.3 ~ 30 PIP_2_/s, with $\Omega=0.2\text{nm}^{-1}$ for a micelle [10]. This yields *k*_2_ ~ 1.5-150 (nm)/s.

**3**. As the membrane bends, the local curvature increases, and so does the exposure of the polar head of PIP_2_ to enzymes. The curvature-mediated exposure of PIP_2_ enhances PIP_2_ phosphatase recruitment, and can be inferred from *in vitro* measurements.

The curvature-dependent factor for phosphatase recruitment rate, α, has units of length (nm); it defines the threshold membrane curvature (1/α in Eq. [2]), above which the enzyme recruitment is rapidly enhanced. Such ‘geometric fitting’ would necessarily translate into a decrease in the energy barrier for binding, and hence can be included as a Boltzmann factor. This principle is quite general; a similar dependence is also observed in phospholipase A2 and C [12,13]. The value of α can be obtained in at least two ways. (1). α can be measured from the different binding affinities of the enzymes to phosphoinositide-coated lipid vesicles of different sizes [11]. Since the bare lipase enzyme activity in the *in vitro* experiments is extremely low [11], the difference in the apparent phosphoinositol hydrolysis rates on vesicles of different sizes can be attributed to the different binding rate of the lipase. This leads to α ~ 100 nm (see Figure 2 in [11]). (2). The recruitment of enzyme to the membrane usually involves the insertion of the molecule into the bilayer prior to activation. This requires work against the surface pressure ($P_{S}$) of the bilayer, and introduces the Boltzmann factor into its effective recruitment rate. *A* is the surface area per phosphatase or lipase molecule [13]. Membrane curvature reduces the surface pressure by ~ $2{pP}_{S}\sqrt{A}\Omega$ (from Young’s relation: pressure difference = surface tension/membrane curvature), where A is the area of the molecule, and $P_{S}\sqrt{A}$ is the effective surface tension of the membrane. This factor effectively increases the recruitment rate of phosphatase by . From this we obtain . The surface pressure of lipid bilayer $P_{S}$ ~ 10-100 mN/m [14,15], and the linear dimension of the enzyme is ~ 1 nm, which yields .

**4**. From Michaelis-Menten kinetics, the Michaelis constant is , where $k_{4}$is the enzyme turnover rate of enzyme, $k_{2}\Omega$ is the apparent hydrolysis rate, and $k_{3}e^{\alpha\Omega}$is the enzyme recruitment rate. We obtain the values of *k*_3_ and *k*_4_ from the Michaelis constant measurements on liposomes of different sizes [11]. From above derivation, *k*_2_ ~ 1.5-150 (nm)/s and *α* ~ 100 nm. In reference [11], for vesicle of ~50 nm in diameter, $K_{M}=0.77\text{mM}$; for vesicles of ~100 nm, $K_{M}=0.3\text{mM}$. We thus obtain *k*_3_ ~ 0.005-0.5/s and *k*_4_ ~ 0.06-6.0/s. We can also obtain the values of *k*_3_ and *k*_4_ by fitting the measurements of binding affinity between phospholipase C and PIP_2_ [11]. If we choose $\alpha=100\text{nm}$, then *k*_3_ ~ 0.15/s and *k*_4_ ~ 0.5/s.

(Note that, the concentration units must be converted into number of molecules: for a vesicle 50 nm in diameter fully covered by PI/PIP_2_, the effective concentration of PI is . So *K*_M_ = 0.77 mM would corresponds to a PI coverage fraction ~ 0. 385%. A similar derivation for a vesicle diameter of ~ 100nm yields that $K_{M}=0.3\text{mM}$ corresponds to the PI coverage fraction ~ 0.3%.)

**5**. We attribute the enhanced turnover rate of coat proteins to the mismatch between its preferred curvature and the mean curvature of the bud membrane. This effectively includes all the factors that contribute to the coat protein disassembly. Such an overall effect should also include the curvature-dependent GTPase activity that is responsible for actively disassembling the coat protein. The sensitivity parameter, β, can be inferred from measurements of COPI disassembly rate on liposomes of different diameters [16]. We assume that COPI and clathrin are similar in their disassembly processes. This is because Arf GTPase has been shown to regulate both the disassembly of the COPI in mammalian COPI-mediated vesicular membrane trafficking and that of the coat proteins in clathrin-mediated endocytosis [16,17]. Here, we use the spontaneous curvature of clathrin as the preferred curvature in Eq. [3], which is ~ corresponding to a vesicle diameter of ~ 60-100 nm [18]. Experiments show ~ 50 times slower coat protein disassembly when the vesicle size increases from 50 nm to 80 nm [16]. Setting the spontaneous curvature as in Eq. [3], we obtain β ~ 2700−40000 nm^2^.

**6**. The sensitivity parameter, χ, can be derived from BDP binding experiments on different vesicle sizes [19]. Similar to the derivations for α and β, this yields χ ~ 100-1000nm^2^.

**7**. In the model, the coat protein recruitment rate is$k_{5}P/(1+K_{1}P)$. We assume the adaptor proteins quickly reach their equilibrium in binding to PIP_2_ with affinity 1/*K*_1_, which then recruit the coat proteins to the bud region with apparent rate *k*_5_. FRAP experiments show that the typical recovery time of clathrin at an endocytic site is t_1/2_ ~ 10 - 20 seconds [20,21,22]. These measurements suggests that the apparent recruitment rate of the coat protein is ~ 0.05-0.1/s. In the calculations, we choose $k_{5}$ to ensure that the recruitment rate at its maximum level is 0.05-0.1/s and, hence, we obtain $k_{5}$ ~ 0.1-0.2/s. This is because, the PIP_2_ coverage fraction at PM would increase at the endocytic site, such that will approach 0.5 (given that *K*_1_ ~1.0 μM^-1^). This introduces a factor of two-fold into the apparent recruitment rate to account for the effective rate 0.05-0.1/s. This leads to $k_{5}$ in the range of 0.1-0.2/s. The coat protein turnover rate is inferred from its disassembly rate during the un-coating process, whose t_1/2_ is about 2-5 seconds [23]. This sets the turnover rate $k_{6}$~ 0.2-0.5/s.

**8**. The functional module **A** in the model represents the local F-actin level on the membrane surface. Actin polymerization rates are typically ~45 nm/s [24,25], which corresponds to the addition of 16 actin subunits/s per linker/nucleator to the membrane surface. In the model, the linker of F-actin is the functional module **C** (coat protein). Taking this discrete reaction event into the continuous scheme, the apparent F-actin recruitment rate is $k_{7}C$, where *k_7_* in the model used for numerical calculation is . This assumes that an actin subunit has a rod-like shape 2.7nm in length and ~ 5nm in diameter. The addition of actins is distributed over the surface area, which is re-scaled by the unit arc length 1nms. Since F-actin polymerization at the endocytic site advances its filament front ~ 150 nm in about 10 seconds [24,25], its depolymerization rate is ~ 30 nm/s, given that its polymerization rate is ~ 45nm/s. Accordingly, the typical value for the turnover rate used in the model is *k_8_* ~ $0.42/s$.

Also, the force generation process of actin and myosin activity is typically cooperative and highly nonlinear: it may remain at basal level when the F-actin level is below a certain threshold, *A*_0_, above which it sharply increases, resulting in a nearly step-wise function. To capture the simplest scenario, the effective pulling force at the bud region is taken as: where *f_0_* is the force constant, and *A*_0_ = 0.5. The choice of *A*_0_ and the specific form of the effective force does not affect the qualitative results.

**9**. In the model, BDPs protect its underlying PIP_2_ by attenuating the hydrolysis rate by a factor of $(1+K_{2}B)$. This protection effect is due to the tight binding between PIP_2_ and BDPs that leaves little room for phosphatase access. Such protection strength, *K_2_*, is equal to the inverse of the dissociation constant between PIP_2_ and the PH-domain proteins [26,27,28], the ENTH-domain protein such as epsin [28], and the EFC-domain PCH protein, all of which show weak homology to BDP [28]. These measurements yield *K_2_* ~ 0.6-10.0 μM^-1^.

**10.** We neglected diffusion of the endocytic proteins in our model. The reasoning is as follows.

The chemical reactions on the endocytic membrane typically involve three sub-processes: (a) reactant association with the membrane; (b) the reaction on the membrane; (c) reactant dissociation from the membrane. The sub-processes (a) and (c) involve reactant diffusion in 3 dimensions (i.e. in solution), while the sub-process (b) involve diffusion in 2-D (i.e. on the membrane surface).

(1) We assume that the available pools of these proteins in 3-D (inside the cell) are infinitely large reservoirs as far as endocytosis is concerned. That is, the assembly/ disassembly reactions on the membrane, determined by specificity, are not limited by diffusion in 3-D. Indeed, the reaction rates used in the model are the effective rates taken or deduced from experimental measurements (e.g. FRAP), which already include the effects of 3-D diffusion.

(2). The reasons for neglecting 2-D diffusion on the membrane (sub-process (b)) are as follows. For the coat proteins the bud and the BDPs on the tubule, their 2-D diffusion include the diffusion of the assembled forms and their monomers. The monomer diffusion is part of the turnover processes of the reactions measured by FRAP, from which we deduced the reaction rates. Thus their effects have already been absorbed into their respective turnover rates. Moreover, the coat proteins and the BDPs form large scaffolds of higher order structures: i.e., clathrin forms a regular lattice at the bud, and BDPs forms spirals wrapping around the membrane tubule. These structures hardly diffuse due to their large size and/or their high binding affinity to the membrane. Consequently, their diffusion is neglected in the model.

(3). Because the large scaffolds of coat proteins and BDPs are not expected to diffuse appreciably, their bound PIP_2_ in the membrane would not diffuse much either, as long as the binding affinity is high enough to dominate diffusion. The PIP_2_ diffusion rate in the plasma membrane has been measured as ~ $1{{mm}^{2}}/s$ [29]. According to our estimate, to prevent PIP_2_ diffusion requires only a binding affinity of BDPs to the coat protein in the micro-molar range, which is well within their measured affinity. Thus, PIP_2_ diffusion is neglected in the model. It follows that the phosphatase that binds to PIP_2_ cannot diffuse much in 2-D.

(4). In the model, we treated the actin functional module as an effective force on the membrane surface of the bud, and did not explicitly represent its geometrical structure. As we argued above, 3-D diffusion of actin has been implicitly embodied in the assembly and the disassembly rates and, hence, is neglected in the equations. On the 2-D membrane surface, the actin filaments cannot diffuse much, as they are organized by and firmly bind to the scaffold of the coat proteins at the bud that has little diffusion. Thus, we neglect the 2-D diffusion of the actin module in the model.

**11**. We neglected the hydrodynamics of the in-plane flows of the membrane constituents in the model. The justification is as follows. Hydrodynamic flow becomes important only when the convection dominates over diffusion of the membrane constituents. The Péclet number *Pe = LV/D* determines when this is valid. Here, *L* is the characteristic length of the system, *V* is the velocity, and *D* is the diffusion constant. When *Pe* is >>1, convection dominates; when *Pe* <<1, hydrodynamics can be neglected. For endocytic membrane deformation in yeast and also similarly in mammalian cells: (1) the characteristic length of the system is the membrane invagination length *L* ~ 200 nm; (2) the maximum convection velocity can be deduced from the measurement of the endocytic membrane tip position over time, which yields *V* ~ 200 nm/5s = 40nm/s; (3) the typical lipid diffusion constant is *D* , and that for a typical integral membrane protein is *D* [29,30]. Under these conditions, the typical Péclet number *Pe* = 0.0008 - 0.008. We thus ignored the hydrodynamics of the membrane constituents in describing the membrane dynamics (Eq. [6] in the model). We modeled the membrane as an elastic sheet, which implies that membrane deformations can be readily accommodated by local re-arrangement of membrane constituents on a much shorter time scale.

**12**. The relative timescale of the membrane dynamics with regard to that of the local chemical reactions is represented by the factor $\Gamma$. The intrinsic timescale of membrane shape change typically correlates as . Here *L* ~ 1 μm is the size of the membrane deformation, and *D* is the effective diffusion coefficient of the membrane constituents, which include lipid and membrane protein. Here, the limiting factor is determined by the slower diffusion of membrane proteins. Thus, the effective diffusion constant *D* shall be in the range of membrane protein diffusion ~ 1.0 ${{mm}^{2}}/s$[29,30]. Therefore, the intrinsic timescale of membrane deformation is , which sets the relative timescale of membrane deformation Γ ~ 1. But what if the relative time scale $G$ is not close to unity? We calculated a phase diagram to see how endocytosis depends on the relative time scale, $G$, and the interfacial force (see Figure S3 and the detailed discussion there).

**13**. The interfacial force, *λ*, is proportional to the difference in PIP_2_ levels across the lipid phase boundary: . Thus the maximum interfacial force, *λ_0_*, is the proportionality factor. It could depend on several factors; we list two.

(1). PIP_2_ hydrolysis on the bud eliminates hydrogen bonds that bridge the interfacial boundary. When this hydrogen bond shielding is lost the boundary is exposed to water, which is energetically unfavorable. The resulting line tension is proportional to the PIP_2_ concentration difference across the interface. The interface tries to contract so as to minimize the unfavorable contacts thus squeezing the neck. We estimated the maximum line tension on the inset to Figure 1A in [31]. After adding 250mM NaCl, the lateral pressure of PIP_2_ monolayer increases by about 15 mN/m. We attribute this increase predominantly to the disruption of hydrogen bonding between PIP_2_ lipids. Therefore the effective contraction force from hydrogen bonding is . (the height of PIP_2_ leaflet ~ 2~3 nm).

(2). The reduced hydrogen bond network on the bud lowers the membrane surface tension of the outer leaflet, thus the outer leaflet tends to expand. This amounts to an increased a lateral surface pressure that propagates from high-pressure region towards the interfacial boundary. Due to the local concavity of the membrane created by the initial interfacial tension, this lateral pressure creates an inwards-directed force at the phase boundary and provides an additional pinching force. This additional lateral pressure also increases with the difference in PIP_2_ levels across the phase boundary. We inferred the maximum force arising from lateral pressure from Figure 1B in [32]. With fixed area per lipid ~ 75 Å^2^ (the physiological value of PIP_2_) which corresponds to a lateral pressure ~ 30mN/m [33], so that the lateral pressure drop due to hydrolyzing PIP2 to PI is ~ 15mN/m. This lateral pressure drop comes from the combined effects of the lateral pressure increase from the disruption of the hydrogen bonding of PIP2 and the lateral pressure drop due to the polar head removal from PIP2. The lateral pressure increase of (a) is ~ 15mN/m. Therefore the lateral pressure drops in (b) shall be 30mN/m. The maximum force from such lateral pressure drop is . The total squeezing force is the sum of the line tension and the additional force from lateral pressure. Therefore, the maximum interfacial force should be in the range of 30 - 135pN.

**14.** We propose BDPs protect the PIP2 from hydrolysis at the tubule more effectively than that of coat proteins at the bud. The reasons are as follows:

(1) The BDP binding affinity to PIP_2_ (< μM) is typically higher than those of ENTH/ANTH domain coat proteins (~several μM) [34,35].

(2) The clathrin coat protein typically forms a meshwork over the bud [18]. The meshwork is very sparse, leaving the majority of the membrane underneath unprotected. This is in sharp contrast to the protection BDP affords PIP2. Experiments demonstrate that BDPs form dense spirals around the membrane tubule surface with pitches of only a few nanometers [36,37,38,39,40,41]. Thus, the denser spiral of BDPs prevents enzyme access to PIP_2_ more effectively than the sparse clathrin meshwork.

(3) Therefore, we assume that the coat proteins protect the PIP_2_ less at the bud than BDP protect PIP_2_ at the tubule, and so a line tension forms at the boundary. If, however, the converse were true (i.e. the bud were more protected than the tubule), then although the resulting interfacial force might be sufficient to drive vesicle scission, there are at least three problems with this scenario that invalidate it. First, since the PIP_2_ level at the bud remains high up to the point that the vesicle gets pinched, the PIP_2_-bound coat proteins would remain at high level as well, which contradicts the experimental observation that the coat proteins decay rapidly upon vesicle scission [25]. Second, as the coat proteins organize the actin/myosin module, the high level of coat proteins facilitates a large force on the bud that opposes the elastic retraction of the membrane. This would may prevent retraction of the membrane invagination concurrent with vesicle scission, which is also inconsistent with experimental observations [25]. Finally, endocytosis is a coherent process: the previous step must pave the way for the next one. The coat proteins at the bud have to disassemble with or shortly after vesicle scission, a step that is essential for the fusion of the endocytic vesicle with early endosomes and their own recycling. If the coat proteins bind to PIP_2_ too tightly at the bud, it would become very difficult to disassemble later on and fuse with early endosomes. From the perspective of the coherent process of endocytosis, the proposal that the coat proteins protect the PIP_2_ more potently at the bud appears too uncertain and inefficient.

**15.** For simplicity, the location of the interfacial boundary along the contour length of the membrane has been fixed. Varying the location of the interfacial boundary changes the size of the resulting pinched vesicle. The endocytic vesicle in yeast is ~ 50 nm in diameter, and our choice for the interfacial boundary in the model is located so as to produce this size. It is possible that the endocytic cargo helps define the size of the vesicle and hence defines the phase boundary. In the later stage of endocytic vesicle formation (the last 30 seconds of the total 2-minute endocytic process), cargo is expected to fully occupy the membrane leaflet facing the extracellular environment. Hence, the interfacial boundary is pre-determined in the model.

# B. Table 1. Model Parameters

| **Symbol** | **Parameter Description** | **Measured**  **Value** | **Value used in the fitting** [*] | **Reference** |
| --- | --- | --- | --- | --- |
| $k_{1}$ | PIP_2_ synthesis rate | ~0.4-7.0/s | 0.7/s | [2,3,4] |
|  | Curvature-dependent PIP_2_ hydrolysis rate | ~1.5-150(nm)/s | 20(nm)/s | [5,6,7,8,9] |
|  | Enzyme recruitment rate | ~0.005-0.5/s | 0.2/s | [11] |
|  | Enzyme turnover rate | ~0.06-6.0/s | 0.6/s | [11] |
|  | Recruitment rate of coat protein | ~0.1-0.2/s | 0.15/s | [20,21,22] |
|  | Turnover rate of coat protein | ~0.2-0.5/s | 0.40/s | [23] |
|  | Assembly rate of F-actin | ~45nm/s | 45nm/s | [24,25] |
|  | Disassembly rate of F-actin | ~30nm/s | 30nm/s | [24,25] |
|  | Intrinsic BDP recruitment rate | Estimate | 1.2/s |  |
|  | Actin-dependent BDP recruitment rate | Estimate | 100.0/s ^[**]^ |  |
|  | Disassembly rate of BDP | Estimate | 1.5/s |  |
|  | The relative timescale of BDP dynamics | 1.0 | 1.0 |  |
|  | Affinity between PIP_2_ and the adaptor protein that recruits PIP5K | ~ 0.5-2.0μM^-1^ | 1.0 μM^-1^ | [1] |
|  | Protection strength of BDP against PIP_2_ hydrolysis | ~ 0.6-10.0μM^-1^ | 1.0 μM^-1^ | [26,27,28] |
| *α* | Curvature-sensitivity factor of enzyme recruitment rate | ~ 30-300 nm | 100 nm | [11,13,14,15] |
| *β* | Curvature-sensitivity factor of coat protein turnover rate | ~- nm^2^ | 7000 nm^2^ | [16] |
| *χ* | Curvature-sensitivity factor of BDP recruitment rate | ~10^2^-10^3^nm^2^ | 500 nm^2^ | [19] |
|  | Relative timescale of the membrane dynamics | ~1.0 | 1.0 | [30] |
| *κ* | Membrane bending modulus | ~100*k*_B_T | 100*k*_B_T | [42,43] |
| *σ* | Membrane surface tension | 10^-4^~10^-5^ N/m | 10^-4^ N/m | [42,43] |
|  | The preferred curvature of coat protein at the bud |  |  | [16] |
|  | The preferred curvature of BDP at the tubule | ~ |  | [19,36,37,38,39,40,41] |
| *λ*_0_ | The maximum interfacial force from lipid phase segregation | 10-100pN | 50pN | [31,32,44,45] |
| *f*_0_ | The contractile force from F-actin and myosin activity | ~1.0-4.0pN | 2.0pN | [46,47] |
| *n* | The Hill coefficient of BAR binding to PIP_2_ |  | 3 | [19,48,49] |
|  | Osmotic pressure | ~1000 Pa | 0 ^[***]^ | [50] |

[*] The values are used for fitting to the experimental time-lapse data. If not otherwise specified, they are fixed throughout the paper.

[**] During the course of endocytosis predicted by our model, the average actin coverage fraction at the endocytic site is calculated from . From this, the typical maximum value during endocytosis $\bar{A}$ ~ 0.2. Thus, the maximum actin-aided BDP recruitment rate is ~ 4 per second; this is of the same order of magnitude as the kinetic parameters of other functional modules.

[***] It can be shown that the effective pressure impinging upon the endocytic membrane generated from F-actin and myosin motor activity is much larger than the osmotic pressure, so we omit it in our calculations, see [51].

# C. Membrane Free Energy Functional

The free energy functional for the endocytic membrane (the right hand side of equation 6 in the text) is given by:

(C1)

Equation C1 consists of the contributions from three regions along the membrane invagination: (i) the bud region, (ii) the tubule region, and (iii) the interface boundary between the bud and the tubule. We assume that the endocytic membrane has cylindrical symmetry. The shape at each location along the arc length *s* is uniquely defined by the tangent angle, $j(s)$, and the radius, $r(s)$. is the local membrane curvature in the tangential direction, and $\text{sin}j(s)/r(s)$ is the local curvature in the orthogonal radial direction. The local mean curvature is and the Gaussian curvature is . In Eq. (C1), *κ*, *κ*_G_, and *σ* are the bending modulus, Gaussian bending modulus, and surface tension of the membrane, respectively. $j_{0}$ is the average angle between the base and the F-actin (at which the impinging forces pull the bud membrane) ~ 120^0^; this could vary for each actin filament, or over time. We have ignored these complications in this study. *a* ~ 5nm is the diameter of an actin filament; and *f* is the pulling force from actin module. and are the spontaneous curvatures preferred by the coat proteins in the bud and BDPs in the tubule, respectively. Note that, the preferred curvature at the bud region stems from coat proteins, e.g. clathrin, which encage the vesicle and, hence, affects the mean curvature:. As BDPs tubulate the membrane, they would only affect the membrane curvature in the radial direction:$\text{sin}j(s)/r(s)$. The interfacial force depends on the difference in local PIP_2_ level across the interface .

# D. Membrane tubulation driven by BDPs binding

## Qualitative Description

As BDPs bind to the flat surface of a lipid bilayer, they tend to deform its the membrane to conform to their intrinsic curvature. The bending force imposed on the membrane by the BDPs is opposed by the membrane elasticity, which prefers to remain flat. On the one hand, a BDP does not bind as well to a membrane whose local curvature does not fit its preferred shape. Weaker binding is reflected in its reduced binding rate or increased turnover rate as compared with a perfect match. At the same time, bending of the local membrane imposed by the BDPs affects the membrane shape nearby so that the BDP binding rate is higher than on a flat membrane. Therefore, once a BDP binds to the membrane, it increases the binding rate in the surrounding membrane. As more BDPs bind to the membrane, it will come to dominate the intrinsic energy penalty incurred by membrane bending, and impose its own local curvature on the membrane. This further stabilizes its binding and facilitates their subsequent binding at the membrane nearby. This is a positive feedback loop, and eventually the flat membrane will be tubulated by BDPs.

## Mathematical Description

The dynamics of the protein recruitment/turnover and the underlying membrane shape change can be described by the following equations, where the BDPs are treated as a continuum mean field quantity.

*Dynamics of BDPs on the membrane:*

(D1)

*Dynamics of the membrane shape change:*

(D2)

In Eq. (D1), 0 ≤ *B*(*s*) ≤ 1 is the local coverage fraction of BDP, and *s* is the coordinate along the membrane surface. The exponential term ensures that the recruitment rate (i.e. *k*_on_) will be maximum if the local membrane curvature in the radial direction, $\Omega(s)$, perfectly fits the preferred shape of the BDP, $\Omega_{B}^{(0)}$, when it fully covers the local area (*B*(*s*) = 1).

The membrane shape is dictated by the local coverage of BDPs. In Eq. (D2), *F* is the elastic energy of the membrane (see Eq. (D3)), $t_{M}$is the relative timescale of the membrane dynamics with regard to BDP dynamics. The notations in the Eqs (D1-3) are the same as in the main text, and the parameter values are listed in **Table 1** of **Section B**.

(D3)

## The dynamical evolution of the system

The dynamics of the system can be obtained by integrating Eqs. (D1-3) numerically over time. Figure S1 shows the growth of a long tubule from a patch of membrane of 500 nm in radius.

# E. Additional phase diagrams

# (I) The effects of curvature-independent PIP_2_ hydrolysis on endocytosis in budding yeast

We explore the effects of curvature-independent PIP_2_ hydrolysis on endocytosis. To that end, we make a small modification of the model. We split the PIP_2_ hydrolysis rate into two parts; one ($k_{2}\Omega(s)$) depends on the local membrane curvature while the other ($k_{2}^{(0)}$) does not. The equations for the PIP_2_ dynamics become:

PIP_2_ dynamics in the bud region:

(E1)

PIP_2_ dynamics in the neck region:

(E2)

Figure S2 is a phase diagram for endocytosis computed from this revised model, which is characterized by the curvature-dependent and curvature-independent PIP_2_ hydrolysis rates. It shows that successful endocytosis (*i.e*. vesicle scission) absolutely requires curvature-dependent PIP_2_ hydrolysis. Otherwise, endocytosis cannot be successful no matter how fast is the absolute rate of curvature-independent PIP_2_ hydrolysis. In the latter scenario, the interfacial force barely develops (data not shown). This finding underscores the importance of the positive feedback loop between the development of interfacial force and curvature-dependent PIP_2_ hydrolysis for rapid and timely vesicle scission.

# (II) The effects of relative timescale of membrane dynamics on endocytosis in budding yeast

We carried out a phase diagram calculation to investigate how endocytosis depends on the relative time scale, $G$ ,and the interfacial force. As Figure S3 shows, when the membrane dynamics is much faster than that of chemical reactions ($G>1$), the membrane can quickly relax to its equilibrium shape. With increasing $G$, the required interfacial force constant, $\lambda_{0}$, for successful endocytosis asymptotically approximates the one obtained from an equilibrium calculation ~ 50 - 60pN; i.e. in which the equilibrium membrane shape is obtained from the minimization of the Helfrich membrane free energy previously calculated [51]. On the other hand, when the membrane shape change is much slower than the chemical reactions ($G<1$), endocytosis can still be successful but requires a much larger interfacial force constant $\lambda_{0}$. This is because, a slow membrane response requires a faster interfacial force response (larger$\lambda_{0}$) and, hence, a larger interfacial force for a given PIP_2_ concentration difference across the interfacial boundary to invoke its positive feedback with the curvature-dependent PIP_2_ hydrolysis. At $G=1$, the required $\lambda_{0}$ for successful endocytosis is minimal; in other words, endocytosis under this condition is the easiest.

Note that the relative timescale of membrane dynamics and chemical reactions needs to be comparable in order to invoke the positive feedback between them. This is qualitatively similar to the conclusion drawn from the linear stability analysis on other systems [52,53].

# (III) Phase diagram of endocytosis on PIP_2_ synthesis and phosphatase turnover in budding yeast

Figure S4 is the calculated phase diagram predicting how endocytosis depends on the PIP_2_ synthesis rate and PIP_2_ phosphatase turnover rate. Because PIP_2_ is the basis for endocytic apparatus assembly, a sufficient PIP_2_ level is a prerequisite for successful endocytosis. It is, therefore, easy to understand why there must be a threshold PIP_2_ synthesis rate. When PIP_2_ phosphatase turnover rate is relatively low (say ~ 0.6/s), endocytosis can be realized over a very broad range of PIP_2_ synthesis rate. In this scenario, as long as PIP_2_ can be synthesized fast enough to keep up with phosphatase activity to maintain sufficient PIP_2_ level. No matter how fast PIP_2_ is synthesized the phosphatase activity can always curb the PIP_2_ level increase. Constraining the PIP_2_ level would limit the timescale of endocytic apparatus assembly. As long as the PIP_2_ level is above a threshold, the endocytic apparatus can always be assembled properly and endocytosis can be successful. In fact, the higher the PIP_2_ level, the faster the endocytosis is completed in this limit (data not shown). This prediction is consistent with the general notion that PIP_2_ levels positively regulates endocytosis [54,55,56].

When the PIP_2_ phosphatase turnover rate becomes much faster, the range of PIP_2_ synthesis rate for successful endocytosis is substantially reduced. This is because, when both PIP_2_ phosphatase turnover and PIP_2_ synthesis are too fast, the overall timescale of all the chemical reactions become too fast for the endocytic membrane shape change to keep up. This is in the same limit as the slower timescale of membrane dynamics in Figure S3. As the positive feedback loops between membrane curvature and the chemical reactions cannot be invoked, endocytosis would be impaired. To rescue endocytosis in this limit (very small Γ) would require a larger interfacial force as shown in Figure S3.

# F. The functional module description can account for endocytosis in mammalian cells

Here we use the framework of our model to recapitulate mammalian clathrin-mediated endocytosis. In terms of functional modules, the key differences between mammalian and yeast endocytosis are: (i) BDP’s functional role is replaced by dynamin, and (ii) actin does not appear to be essential in recruiting dynamin to endocytic sites. We can ‘re-wire’ the interaction diagram for the endocytic functional modules as shown in Figure S5.

## Calculated Endocytosis Dynamics in Mammalian Cells

Using the same numerical procedure as in budding yeast, we can obtain the dynamics of clathrin-mediated endocytosis in mammalian cells. Figure S6 shows a typical model prediction.

In the model we propose that local membrane curvature is both slave to, and master over, the accompanying endocytic protein dynamics such that their interplay drives the progression of endocytic vesicle formation. Such curvature-mediated feedback could be a combined effect that incorporates protein-protein interactions [57]. We notice that synaptojanin (PIP_2_ phosphatase) has a proline-rich domain similar to dynamin and binds to many of the same binding partners of dynamin (SH3-domain containing proteins), e.g., amphiphysin and endophilin [58,59,60]. Thus, it is plausible that dynamin and synaptojanin bind to these endocytic proteins mutually exclusively. Interestingly, many of these SH3-domain proteins are recruited by clathrin and adaptor proteins to the endocytic site at very early stages [57]. In addition, they also contain BAR-domain proteins, e.g. amphiphysin, which is capable of curvature sensing and curvature producing [19]. It is likely that these early SH3-domain containing BDPs cooperate with clathrin to help deform the endocytic membrane. Due to its intrinsic curvature sensitivity, dynamin would rather accumulate at the collar of the coated pit than the bud region of the endocytic site. The dynamin module in our current model is really the combination of dynamin and its binding partners (SH3-domain containing BDPs). Dynamin accumulation at the collar of coated pit prevents synaptojanin recruitment there due to their mutual exclusion. In this sense, dynamin protects the PIP_2_ at the tubule region from hydrolysis that gives rise to the initial interfacial force, just as we proposed in the yeast model. Alternatively, dynamin GTPase activity could actively squeeze the membrane tubule, which would effectively act as the initial interfacial force. Either way, the resulting local membrane deformation by dynamin jump-starts the positive feedback between the interfacial force growth and the curvature-dependent PIP_2_ hydrolysis activity, as we showed in the main text. Along with the squeezing effect by the interfacial force, the local membrane curvature deviates from the preferred shape of dynamin, making it ‘unfit’ for dynamin binding and thus speeding up its turnover. This explains the observation that dynamin always disassembles before vesicle scission both in vivo and in vitro [61,62,63]. We emphasize that the curvature-mediated dynamin turnover at endocytic sites does not exclude the cooperative dynamin disassembly upon GTP hydrolysis [64].

An interesting question is why do mammalian cells need the extra level of regulation on the BDPs by dynamin in clathrin-mediated endocytosis? This contrasts with budding yeast in which BDPs alone can faithfully facilitate vesicle scission. We speculate that the answer might lie in the different functions of the actin cytoskeleton in the two types of cells. Budding yeast does not move, so actin ia always abundant for the purpose of endocytosis, whereas actin is co-opted to power the motility of mammalian cells and hence needs extra help from dynamin to complete endocytic vesicle formation. However, it is still not clear exactly how the detailed interactions between dynamin, synaptojanin, and other endocytic proteins, e.g. clathrin, amphiphysin, endophilin and actin, are coordinated in endocytosis. Our current model simply provides a general framework for understanding clathrin-mediated endocytosis in mammalian cells. These detailed issues require further study.

## Actin is Not Essential for Endocytosis in Mammalian Cell

Actin is essential for endocytosis in budding yeast [25], which is recapitulated and explained by our yeast model. Actin is largely dispensable for mammalian cells [61,65], which is captured by the modified model. Figure S7 is the calculated phase diagram for clathrin-mediated endocytosis in mammalian cells, characterized by actin dynamics and dynamin dynamics.

Figure S7 shows that, a sufficient supply of dynamin can ensure that endocytosis proceeds normally even in the absence of actin; this is consistent with experimental observations [66]. On the one hand, the roles of both BDPs and dynamin-GTPase are similar in terms of creating the lipid phase segregation, an interfacial force and vesicle scission. On the other hand, dynamin-GTPase would be much more potent than BDPs: it alone can actively deform the endocytic membrane by making use of the full positive feedback loop between its curvature-sensing and deforming power in mammalian cells [67,68], whereas in budding yeast BDPs have to rely on actin driving out the endocytic membrane tubule to initiate its own assembly process. The potency of dynamin could be due to its utilization of GTPase hydrolysis energy, which could also lead to a faster induction of lipid phase segregation. Thus, actin polymerization could play a more passive role in endocytic membrane deformation of mammalian cells: it can simply follow the membrane tubulation by dynamin, instead of creating membrane shape for BDPs assembly as in budding yeast. This re-wiring of the interaction between dynamin and actin indeed underscores a key difference between mammalian and yeast endocytosis. The lack of actin pulling force and the high potency of dynamin also result in the distinct endocytic membrane shape in mammalian cells. Instead of the elongated tubule in budding yeast [69] [70], the shape of endocytic membrane shape in mammalian cells is a constricted coated pit with a very short neck [61]; this is readily captured in Figure S6.

Figure S7 also shows that, endocytosis can still be successful when dynamin is less active, but actin is hyperactive (e.g., dynamin recruitment rate drops from 4/s to 1/s and actin polymerization rate increases from 22.5nm/s to 45nm/s, respectively). In this case, the primary shape of endocytic membrane changes from a constricted coated pit to a long tubular vesicle, just as in budding yeast (data not shown). This result suggests that membrane shape might not be essential in determining the fate of endocytosis. Instead, it may simply reflect the dynamics of the pulling force from the actin module and the pinching force (involving dynamin). If the pinching force develops much faster than the pulling force, then the vesicle would scission before the membrane is tubulated. Otherwise, the membrane could be significantly tubulated before being pinched off.

Interestingly, Figure S7 also shows that endocytosis is impaired when both dynamin and actin activities are over-expressed. If dynamin persists at the endocytic membrane, it would impose its own preferred shape (a tubule ~ 20nm in diameter), and hence prevent further squeezing, just as t over-expressed BDPs in budding yeast. Although our current model does not explicitly take it into account, this result underscores the functional role of the delicate dynamin-actin interaction in clathrin-mediated endocytosis in mammalian cells [71]. We speculate that such dynamin-actin interactions could be a safe measure taken by cells to ensure successful endocytosis.

## Clathrin Is Essential for Endocytosis in Mammalian Cells

Figure S8 shows that, depleting coat protein at the bud while leaving the location of the boundary intact inhibits endocytosis and leads to a phenotype with greatly reduced dynamin recruitment and no vesicle scission. As clathrin deforms the vesicle region of the endocytic membrane [18], it increases the local membrane curvature making it more fit for dynamin binding along the margin adjacent to the tubule region. Thus, without clathrin, the initiation of dynamin recruitment to the endocytic membrane becomes much more difficult. Consequently, it takes much longer for the positive feedback loop between dynamin recruitment and the local membrane shape to rise to its full strength. During this period, the enzyme that hydrolyzes PIP_2_ becomes dominant, keeping PIP_2_ levels low, further inhibiting dynamin recruitment. Note that, dynamin recruitment is explicitly mediated by the membrane curvature in our model. However, this curvature-mediated effect could include other specific protein-protein interactions [72,73]. Indeed, experiments suggest that (i) amphiphysin directly binds to clathrin at an early stage of endocytosis [74]; (ii) amphiphysin mediates dynamin recruitment to the endocytic site, which in turn replaces clathrin and binds to amphiphysin [73,74]; (iii) amphiphysin-mediated dynamin GTPase activity depends on membrane curvature [73]; (iv) experiments show that SNX9, another BDP, coordinates the interaction between actin, dynamin 2, and PI4P5 kinase. This coordination appears to be essential for clathrin-mediated endocytosis in mammalian cells [75,76,77].

## Proper dynamics of dynamin is critical for endocytosis in mammalian cells

Figure S9 predicts that depleting dynamin inhibits endocytosis with no vesicle scission, consistent with the dynamin knockout phenotype [78]. Typically, clathrin is capable of deforming membrane into its own preferred shape (a small bump < 50nm, [18]). Its accumulation and underlying membrane shape form a positive feedback loop, but there is a key difference here from the scenario for BDPs: a clathrin coat imposes a *spherical* shape on its underlying membrane rather than the tubule shape induced by BDPs. The spherical shape necessitates a larger negative Gaussian curvature region at the margin where the spherical membrane merges with the base membrane compared to the corresponding tubule region. This incurs an extra energy penalty for membrane deformation over that confronting BDPs. With no other factor to overcome this additional energy barrier, clathrin can only deform the membrane up to an unconstricted coat pit (Figure S9). The membrane-deforming power of dynamin provides the extra bending energy: using GTP hydrolysis as its fuel, it constricts the membrane at the bottom of the coated pit. Thus, the formation of a constricted coat pit in vivo entails the cooperation between mechanochemical feedbacks between the membrane shape determined by clathrin and by dynamin.

# References

# 1. Krauss M, Kukhtina V, Pechstein A, Haucke V (2006) Stimulation of phosphatidylinositol kinase type I-mediated phosphatidylinositol (4,5)-bisphosphate synthesis by AP-2{micro}-cargo complexes. Proceedings of the National Academy of Sciences 103: 11934-11939.

# 2. Ishihara H, Shibasaki Y, Kizuki N, Wada T, Yazaki Y, et al. (1998) Type I Phosphatidylinositol-4-phosphate 5-Kinases. CLONING OF THE THIRD ISOFORM AND DELETION/SUBSTITUTION ANALYSIS OF MEMBERS OF THIS NOVEL LIPID KINASE FAMILY. J Biol Chem 273: 8741-8748.

# 3. Perera IY, Davis AJ, Galanopoulou D, Im YJ, Boss WF (2005) Characterization and comparative analysis of Arabidopsis phosphatidylinositol phosphate 5-kinase 10 reveals differences in Arabidopsis and human phosphatidylinositol phosphate kinases. FEBS Letters 579: 3427-3432.

# 4. Nakano-Kobayashi A, Yamazaki M, Unoki T, Hongu T, Murata C, et al. (2007) Role of activation of PIP5Kgamma661 by AP-2 complex in synaptic vesicle endocytosis. EMBO J 26: 1105-1116.

# 5. Homma Y, Imaki J, Nakanishi O, Takenawa T (1988) Isolation and characterization of two different forms of inositol phospholipid-specific phospholipase C from rat brain. J Biol Chem 263: 6592-6598.

# 6. Matzaris M, Jackson SP, Laxminarayan KM, Speed CJ, Mitchell CA (1994) Identification and characterization of the phosphatidylinositol-(4, 5)- bisphosphate 5-phosphatase in human platelets. J Biol Chem 269: 3397-3402.

# 7. Chi Y, Zhou B, Wang W-Q, Chung S-K, Kwon Y-U, et al. (2004) Comparative Mechanistic and Substrate Specificity Study of Inositol Polyphosphate 5-Phosphatase Schizosaccharomyces pombe Synaptojanin and SHIP2. J Biol Chem 279: 44987-44995.

# 8. Ooms LM, McColl BK, Wiradjaja F, Wijayaratnam APW, Gleeson P, et al. (2000) The Yeast Inositol Polyphosphate 5-Phosphatases Inp52p and Inp53p Translocate to Actin Patches following Hyperosmotic Stress: Mechanism for Regulating Phosphatidylinositol 4,5-Bisphosphate at Plasma Membrane Invaginations. Mol Cell Biol 20: 9376-9390.

# 9. Cifuentes ME, Honkanen L, Rebecchi MJ (1993) Proteolytic fragments of phosphoinositide-specific phospholipase C- delta 1. Catalytic and membrane binding properties. J Biol Chem 268: 11586-11593.

# 10. Hirai M, Takizawa T, Yabuki S, Nakata Y, Hirai T, et al. (1996) Salt-dependent phase behaviour of the phosphatidylinositol 4,5-diphosphate–water system. Journal of the Chemical Society, Faraday Transactions 92.

# 11. Ahyayauch H, Villar AV, Alonso A, Goni FM (2005) Modulation of PI-Specific Phospholipase C by Membrane Curvature and Molecular Order. Biochemistry 44: 11592-11600.

# 12. Lehtonen JY, Kinnunen PK (1995) Phospholipase A2 as a mechanosensor. Biophys J 68: 1888-1894.

# 13. Boguslavsky V, Rebecchi M, Morris AJ, Jhon DY, Rhee SG, et al. (1994) Effect of Monolayer Surface Pressure on the Activities of Phosphoinositide-Specific Phospholipase C-.beta.1, -.gamma.1, and -.delta.1. Biochemistry 33: 3032-3037.

# 14. Wolfe DH, Brockman HL (1988) Regulation of the Surface Pressure of Lipid Monolayers and Bilayers by the Activity of Water: Derivation and Application of an Equation of State. Proceedings of the National Academy of Sciences 85: 4285-4289.

# 15. Feng Ss (1999) Interpretation of Mechanochemical Properties of Lipid Bilayer Vesicles from the Equation of State or Pressure-Area Measurement of the Monolayer at the Air-Water or Oil-Water Interface. Langmuir 15: 998-1010.

# 16. Bigay J, Gounon P, Robineau S, Antonny B (2003) Lipid packing sensed by ArfGAP1 couples COPI coat disassembly to membrane bilayer curvature. Nature 426: 563-566.

# 17. Toret CP, Lee L, Sekiya-Kawasaki M, Drubin D (2008) Mutiple pathways regulate endocytic coat disassembly in Saccharomyces cerevisiae for optimal downstream trafficking. Traffic 9: 848-859.

# 18. Heuser J (1980) Three-dimensional visualization of coated vesicle formation in fibroblasts. J Cell Biol 84: 560-583.

# 19. Peter BJ, Kent HM, Mills IG, Vallis Y, Butler PJG, et al. (2004) BAR Domains as Sensors of Membrane Curvature: The Amphiphysin BAR Structure. Science 303: 495-499.

# 20. Hinrichsen L, Meyerholz A, Groos S, Ungewickell EJ (2006) Bending a membrane: How clathrin affects budding. Proceedings of the National Academy of Sciences 103: 8715-8720.

# 21. Wu X, Zhao X, Puertollano R, Bonifacino JS, Eisenberg E, et al. (2003) Adaptor and Clathrin Exchange at the Plasma Membrane and trans-Golgi Network. Mol Biol Cell 14: 516-528.

# 22. Wu X, Zhao X, Baylor L, Kaushal S, Eisenberg E, et al. (2001) Clathrin exchange during clathrin-mediated endocytosis. J Cell Biol 155: 291-300.

# 23. Massol RH, Boll W, Griffin AM, Kirchhausen T (2006) A burst of auxilin recruitment determines the onset of clathrin-coated vesicle uncoating. Proceedings of the National Academy of Sciences 103: 10265-10270.

# 24. Sun Y, Martin AC, Drubin DG (2006) Endocytic Internalization in Budding Yeast Requires Coordinated Actin Nucleation and Myosin Motor Activity. Developmental Cell 11: 33-46.

# 25. Kaksonen M, Sun Y, Drubin DG (2003) A Pathway for Association of Receptors, Adaptors, and Actin during Endocytic Internalization. Cell 115: 475-487.

# 26. Chen RH, Corbalan-Garcia S, Bar-Sagi D (1997) The role of the PH domain in the signal-dependent membrane targeting of Sos. EMBO J 16: 1351-1359.

# 27. Lemmon MA, Ferguson KM, O'Brien R, Sigler PB, Schlessinger J (1995) Specific and High-Affinity Binding of Inositol Phosphates to an Isolated Pleckstrin Homology Domain. Proceedings of the National Academy of Sciences 92: 10472-10476.

# 28. Tsujita K, Suetsugu S, Sasaki N, Furutani M, Oikawa T, et al. (2006) Coordination between the actin cytoskeleton and membrane deformation by a novel membrane tubulation domain of PCH proteins is involved in endocytosis. J Cell Biol 172: 269-279.

# 29. Golebiewska U, Nyako M, Woturski W, Zaitseva I, McLaughlin S (2008) Diffusion Coefficient of Fluorescent Phosphatidylinositol 4,5-bisphosphate in the Plasma Membrane of Cells. Mol Biol Cell 19: 1663-1669.

# 30. Lipowski R, Sackmann E (1995) Structure and Dynamics of Membranes (North–Holland, Amsterdam).

# 31. Levental I, Cebers A, Janmey PA (2008) Combined Electrostatics and Hydrogen Bonding Determine Intermolecular Interactions Between Polyphosphoinositides. J Am Chem Soc 130: 9025-9030.

# 32. Levental I, Janmey PA, Cebers A (2008) Electrostatic Contribution to the Surface Pressure of Charged Monolayers Containing Polyphosphoinositides. Biophys J 95: 1199-1205.

# 33. Demel RA, Geurts van Kessel WSM, Zwaal RFA, Roelofsen B, van Deenen LLM (1975) Relation between various phospholipase actions on human red cell membranes and the interfacial phospholipid pressure in monolayers. Biochimica et Biophysica Acta (BBA) - Biomembranes 406: 97-107.

# 34. Itoh T, Koshiba S, Kigawa T, Kikuchi A, Yokoyama S, et al. (2001) Role of the ENTH Domain in Phosphatidylinositol-4,5-Bisphosphate Binding and Endocytosis. Science 291: 1047-1051.

# 35. Ford MGJ, Pearse BMF, Higgins MK, Vallis Y, Owen DJ, et al. (2001) Simultaneous Binding of PtdIns(4,5)P2 and Clathrin by AP180 in the Nucleation of Clathrin Lattices on Membranes. Science 291: 1051-1055.

# 36. Henne WM, Kent HM, Ford MGJ, Hegde BG, Daumke O, et al. (2007) Structure and Analysis of FCHo2 F-BAR Domain: A Dimerizing and Membrane Recruitment Module that Effects Membrane Curvature. Structure 15: 839-852.

# 37. Frost A, Perera R, Roux A, Spasov K, Destaing O, et al. (2008) Structural Basis of Membrane Invagination by F-BAR Domains. Cell 132: 807-817.

# 38. Antonny B (2006) Membrane deformation by protein coats. Current Opinion in Cell Biology 18: 386-394.

# 39. Shimada A, Niwa H, Tsujita K, Suetsugu S, Nitta K, et al. (2007) Curved EFC/F-BAR-Domain Dimers Are Joined End to End into a Filament for Membrane Invagination in Endocytosis. Cell 129: 761-772.

# 40. Dawson JC, Legg JA, Machesky LM (2006) Bar domain proteins: a role in tubulation, scission and actin assembly in clathrin-mediated endocytosis. Trends in Cell Biology 16: 493-498.

# 41. Weissenhorn W (2005) Crystal Structure of the Endophilin-A1 BAR Domain. Journal of Molecular Biology 351: 653-661.

# 42. Simson R, Wallraff E, Faix J, Niewohner J, Gerisch G, et al. (1998) Membrane bending modulus and adhesion energy of wild-type and mutant cells of Dictyostelium lacking talin or cortexillins. Biophys J 74: 514-522.

# 43. Bruinsma R, Behrisch A, Sackmann E (2000) Adhesive switching of membranes: Experiment and theory. Physical Review E 61: 4253.

# 44. Dan N, Safran SA (1998) Effect of Lipid Characteristics on the Structure of Transmembrane Proteins. Biophys J 75: 1410-1414.

# 45. Lipowsky R (1992) Budding of membranes induced by intramembrane domains. J Phys II France 2: 1825-1840.

# 46. Kovar DR, Pollard TD (2004) From the Cover: Insertional assembly of actin filament barbed ends in association with formins produces piconewton forces. Proceedings of the National Academy of Sciences 101: 14725-14730.

# 47. Finer JT, Simmons RM, Spudich JA (1994) Single myosin molecule mechanics: piconewton forces and nanometre steps. Nature 368: 113-119.

# 48. Zimmerberg J, McLaughlin S (2004) Membrane Curvature: How BAR Domains Bend Bilayers. Current Biology 14: R250-R252.

# 49. Gallop JL, Jao CC, Kent HM, Butler PJG, Evans PR, et al. (2006) Mechanism of endophilin N-BAR domain-mediated membrane curvature. EMBO J 25: 2898-2910.

# 50. Rauch C, Farge E (2000) Endocytosis Switch Controlled by Transmembrane Osmotic Pressure and Phospholipid Number Asymmetry. Biophys J 78: 3036-3047.

# 51. Liu J, Kaksonen M, Drubin DG, Oster G (2006) Endocytic vesicle scission by lipid phase boundary forces. Proceedings of the National Academy of Sciences 103: 10277-10282.

# 52. Girard P, Jülicher F, Prost J (2004) Fluid membranes exchanging material with external reservoirs The European Physical Journal E 14: 387-394.

# 53. Rao M, Sarasij RC (2001) Active Fusion and Fission Processes on a Fluid Membrane. Physical Review Letters 87: 128101.

# 54. Lemmon MA (2008) Membrane recognition by phospholipid-binding domains. Nat Rev Mol Cell Biol 9: 99-111.

# 55. van Meer G, Voelker DR, Feigenson GW (2008) Membrane lipids: where they are and how they behave. Nat Rev Mol Cell Biol 9: 112-124.

# 56. Di Paolo G, De Camilli P (2006) Phosphoinositides in cell regulation and membrane dynamics. Nature 443: 651-657.

# 57. Schmid EM, McMahon HT (2007) Integrating molecular and network biology to decode endocytosis. Nature 448: 883-888.

# 58. Hill E, van der Kaay J, Downes CP, Smythe E (2001) The Role of Dynamin and Its Binding Partners in Coated Pit Invagination and Scission. J Cell Biol 152: 309-324.

# 59. Ringstad N, Nemoto Y, De Camilli P (1997) The SH3p4/Sh3p8/SH3p13 protein family: Binding partners for synaptojanin and dynamin via a Grb2-like Src homology 3‚Äâdomain. Proceedings of the National Academy of Sciences of the United States of America 94: 8569-8574.

# 60. Cestra G, Castagnoli L, Dente L, Minenkova O, Petrelli A, et al. (1999) The SH3 Domains of Endophilin and Amphiphysin Bind to the Proline-rich Region of Synaptojanin 1 at Distinct Sites That Display an Unconventional Binding Specificity. J Biol Chem 274: 32001-32007.

# 61. Merrifield CJ, Feldman ME, Wan L, Almers W (2002) Imaging actin and dynamin recruitment during invagination of single clathrin-coated pits. Nat Cell Biol 4: 691-698.

# 62. Pucadyil TJ, Schmid SL (2008) Real-Time Visualization of Dynamin-Catalyzed Membrane Fission and Vesicle Release. Cell 135: 1263-1275.

# 63. Bashkirov PV, Akimov SA, Evseev AI, Schmid SL, Zimmerberg J, et al. (2008) GTPase Cycle of Dynamin Is Coupled to Membrane Squeeze and Release, Leading to Spontaneous Fission. Cell 135: 1276-1286.

# 64. Sever S, Skoch J, Newmyer S, Ramachandran R, Ko D, et al. (2006) Physical and functional connection between auxilin and dynamin during endocytosis. EMBO J 25: 4163–4174.

# 65. Perrais D, Merrifield CJ (2005) Dynamics of Endocytic Vesicle Creation. Developmental Cell 9: 581-592.

# 66. Yarar D, Waterman-Storer CM, Schmid SL (2005) A Dynamic Actin Cytoskeleton Functions at Multiple Stages of Clathrin-mediated Endocytosis. Mol Biol Cell 16: 964-975.

# 67. Praefcke GJK, McMahon HT (2004) The dynamin superfamily: universal membrane tubulation and fission molecules? Nat Rev Mol Cell Biol 5: 133-147.

# 68. Song BD, Schmid SL (2003) A Molecular Motor or a Regulator? Dynamin's in a Class of Its Own. Biochemistry 42: 1369-1376.

# 69. Mulholland J, Preuss D, Moon A, Wong A, Drubin D, et al. (1994) Ultrastructure of the yeast actin cytoskeleton and its association with the plasma membrane. J Cell Biol 125: 381-391.

# 70. Idrissi F-Z, Grotsch H, Fernandez-Golbano IM, Presciatto-Baschong C, Riezman H, et al. (2008) Distinct acto/myosin-I structures associate with endocytic profiles at the plasma membrane. J Cell Biol 180: 1219-1232.

# 71. Schafer DA, Weed SA, Binns D, Karginov AV, Parsons JT, et al. (2002) Dynamin2 and Cortactin Regulate Actin Assembly and Filament Organization. Current Biology 12: 1852-1857.

# 72. Ramachandran R, Schmid SL (2008) Real-time detection reveals that effectors couple dynamin's GTP-dependent conformational changes to the membrane. EMBO J 27: 27-37.

# 73. Yoshida Y, Kinuta M, Abe T, Liang S, Araki K, et al. (2004) The stimulatory action of amphiphysin on dynamin function is dependent on lipid bilayer curvature. EMBO J 23: 3483–3491.

# 74. McMahon HT, Patrick W, Corrin S (1997) Clathrin interacts specifically with amphiphysin and is displaced by dynamin1. FEBS letters 413: 319-322.

# 75. Soulet F, Yarar D, Leonard M, Schmid SL (2005) SNX9 Regulates Dynamin Assembly and Is Required for Efficient Clathrin-mediated Endocytosis. Mol Biol Cell 16: 2058-2067.

# 76. Yarar D, Waterman-Storer CM, Schmid SL (2007) SNX9 Couples Actin Assembly to Phosphoinositide Signals and Is Required for Membrane Remodeling during Endocytosis. 13: 43-56.

# 77. Shin N, Ahn N, Chang-Ileto B, Park J, Takei K, et al. (2008) SNX9 regulates tubular invagination of the plasma membrane through interaction with actin cytoskeleton and dynamin 2. J Cell Sci 121: 1252-1263.

# 78. Sever S, Damke H, Schmid SL (2000) Dynamin:GTP Controls the Formation of Constricted Coated Pits, the Rate Limiting Step in Clathrin-mediated Endocytosis. J Cell Biol 150: 1137-1148.
