# Supplementary figures and images for: The Mechanochemistry of Endocytosis
Source: PLoS Biol. 2009 Sep 29;7(9):e1000204. doi: 10.1371/journal.pbio.1000204 (PMC2742711; doi:10.1371/journal.pbio.1000204)

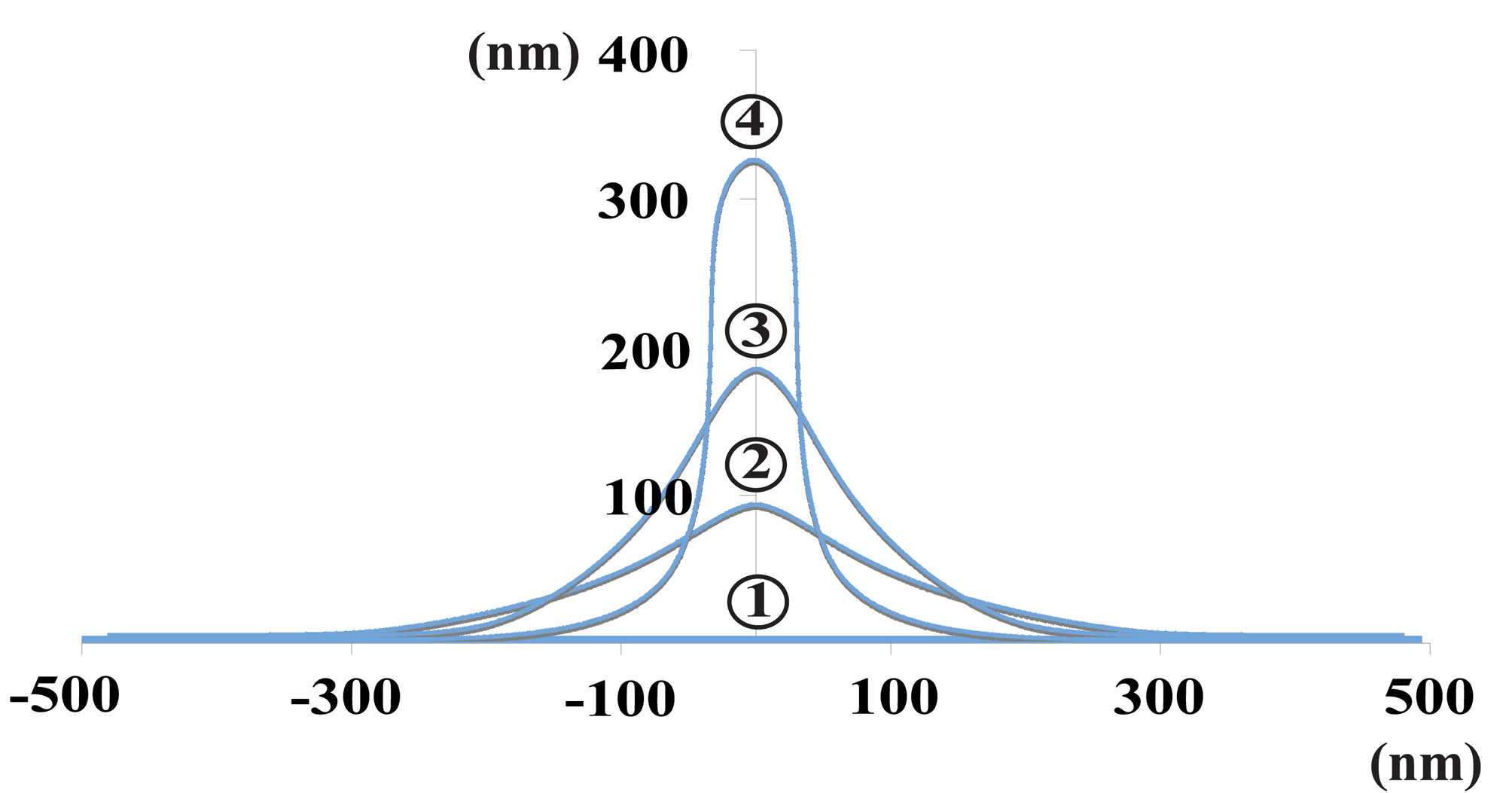

Supplement: Figure S1 — Calculated membrane tubulation driven by BDPs binding. The calculation is carried out in 3-D, and the membrane profile is shown in 2-D. The simulation shows four stages in the growth of the tubule (labeled by time step 1–4). The initial condition is a flat membrane patch, an infinitely large reservoir of BDPs, and there are no membrane-bound BDPs (please see Section D in Protocol S1 for details). (0.23 MB TIF) [file pbio.1000204.s002.tif]

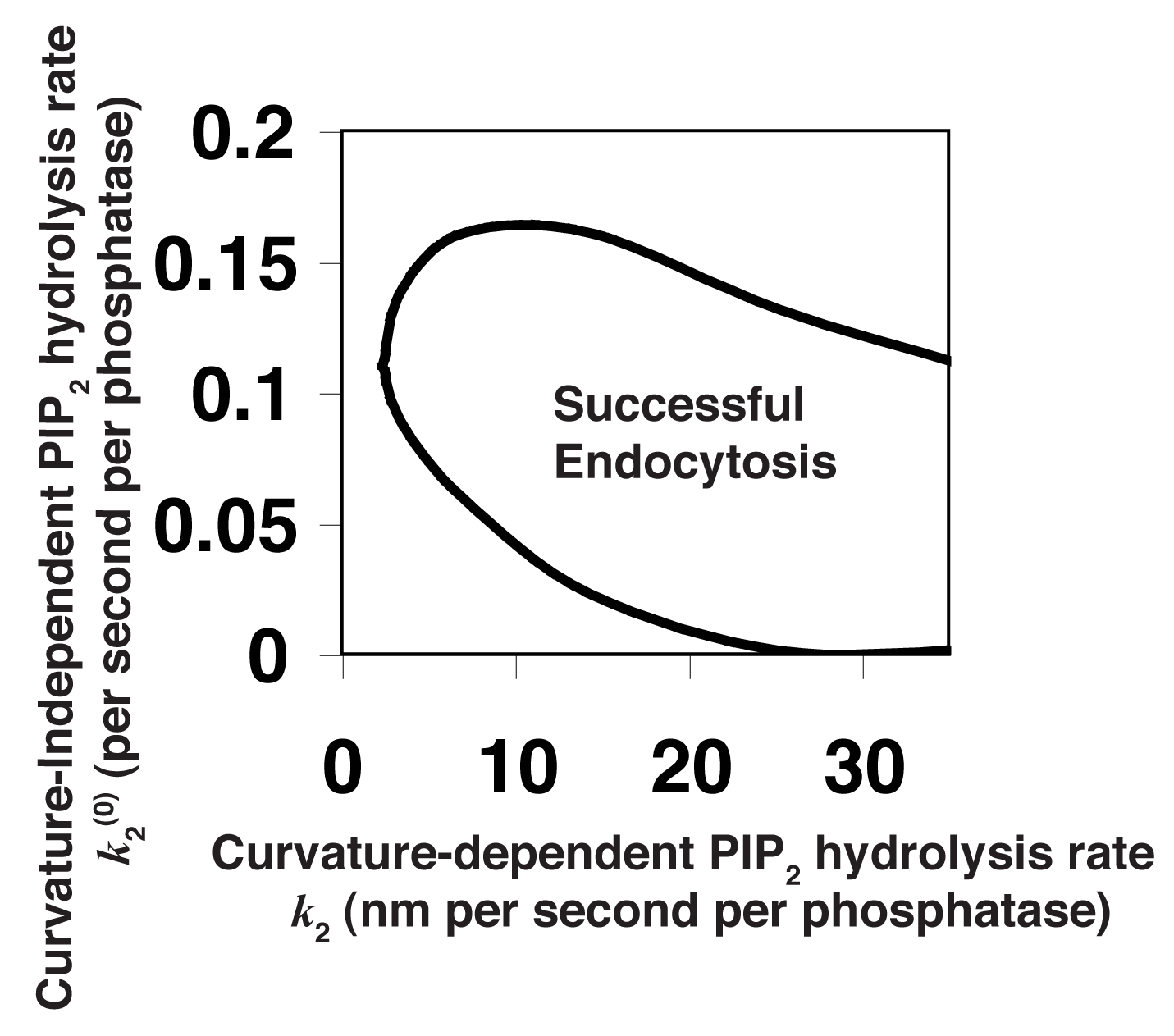

Supplement: Figure S2 — Phase diagram for the fate of endocytosis in budding yeast characterized by the curvature-dependent and the curvature-independent PIP2 hydrolysis rates. Note that since the curvature involved in the membrane invagination is typically ∼1/(100 nm), the range of the curvature-dependent PIP2 hydrolysis rate in Figure S2 is ∼0–0.3 per second per phosphatase that is comparable with (please see Section E(I) in Protocol S1 for details). (0.15 MB TIF) [file pbio.1000204.s003.tif]

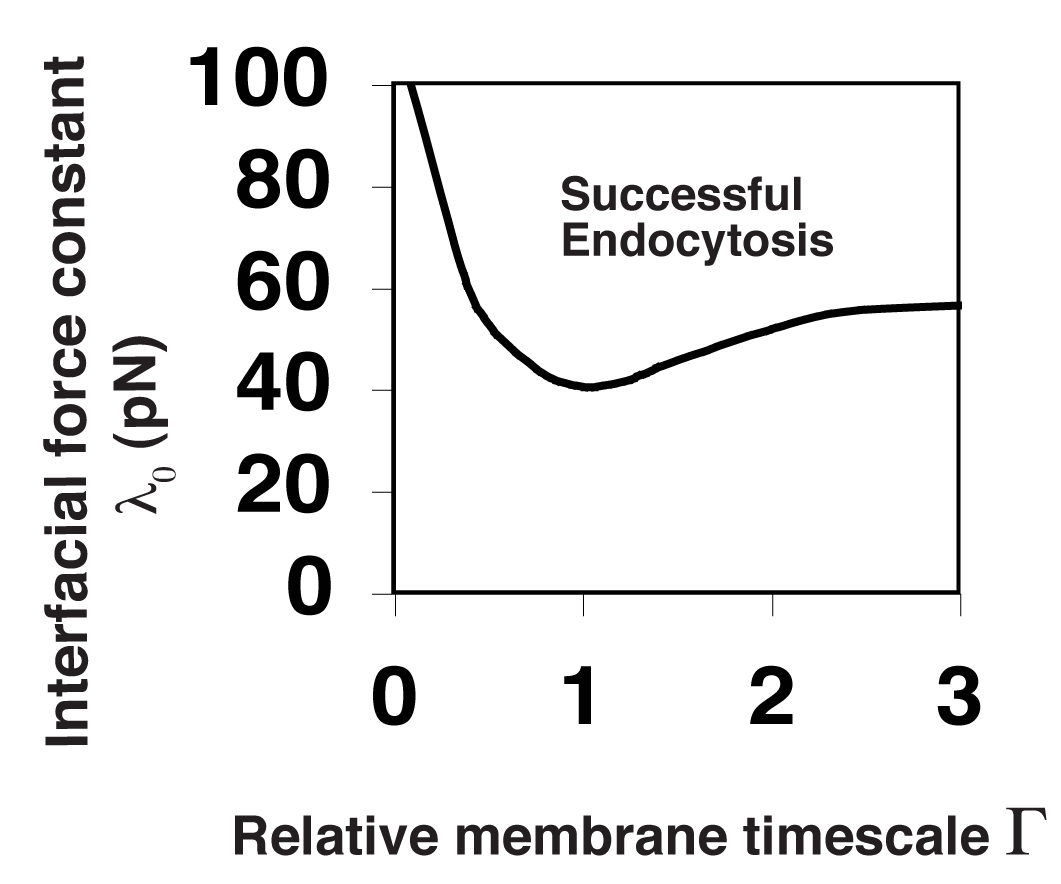

Supplement: Figure S3 — Phase diagram for endocytosis in budding yeast characterized by the relative timescale of membrane dynamics and the interfacial force constant (please see Section E(II) in Protocol S1 for details). (0.10 MB TIF) [file pbio.1000204.s004.tif]

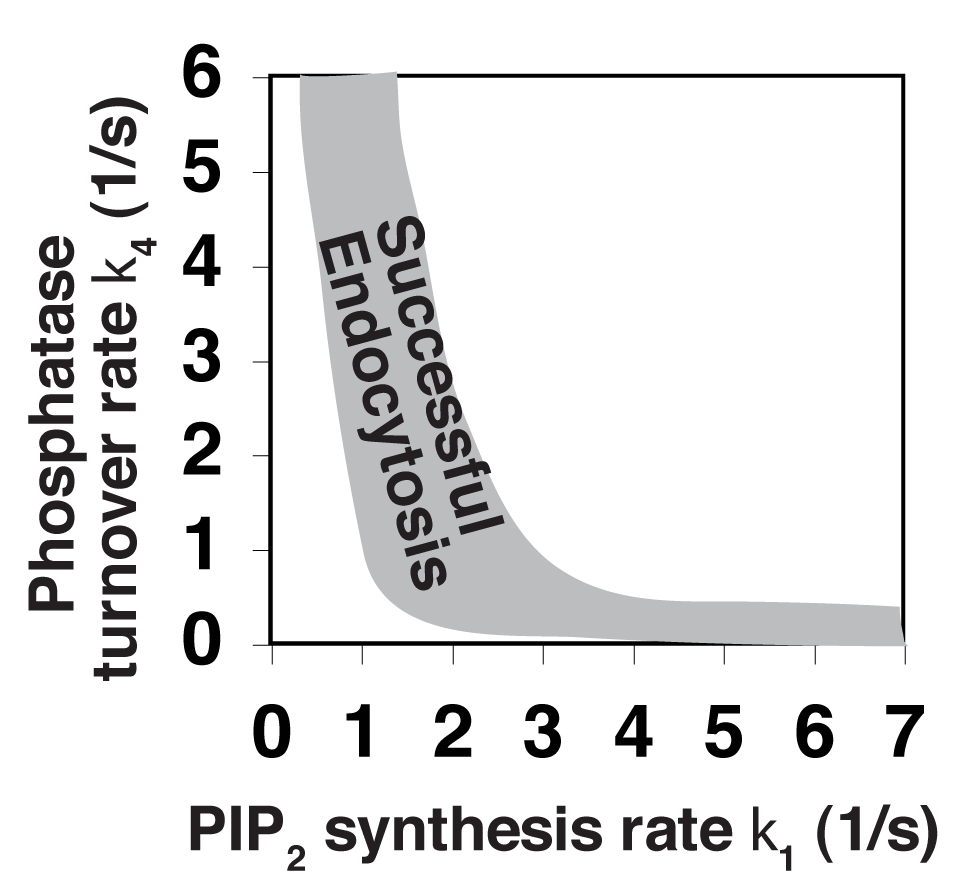

Supplement: Figure S4 — Phase diagram for the fate of endocytosis in budding yeast characterized by PIP2 synthesis rate and PIP2 phosphatase turnover rate (please see Section E(III) in Protocol S1 for details). (0.10 MB TIF) [file pbio.1000204.s005.tif]

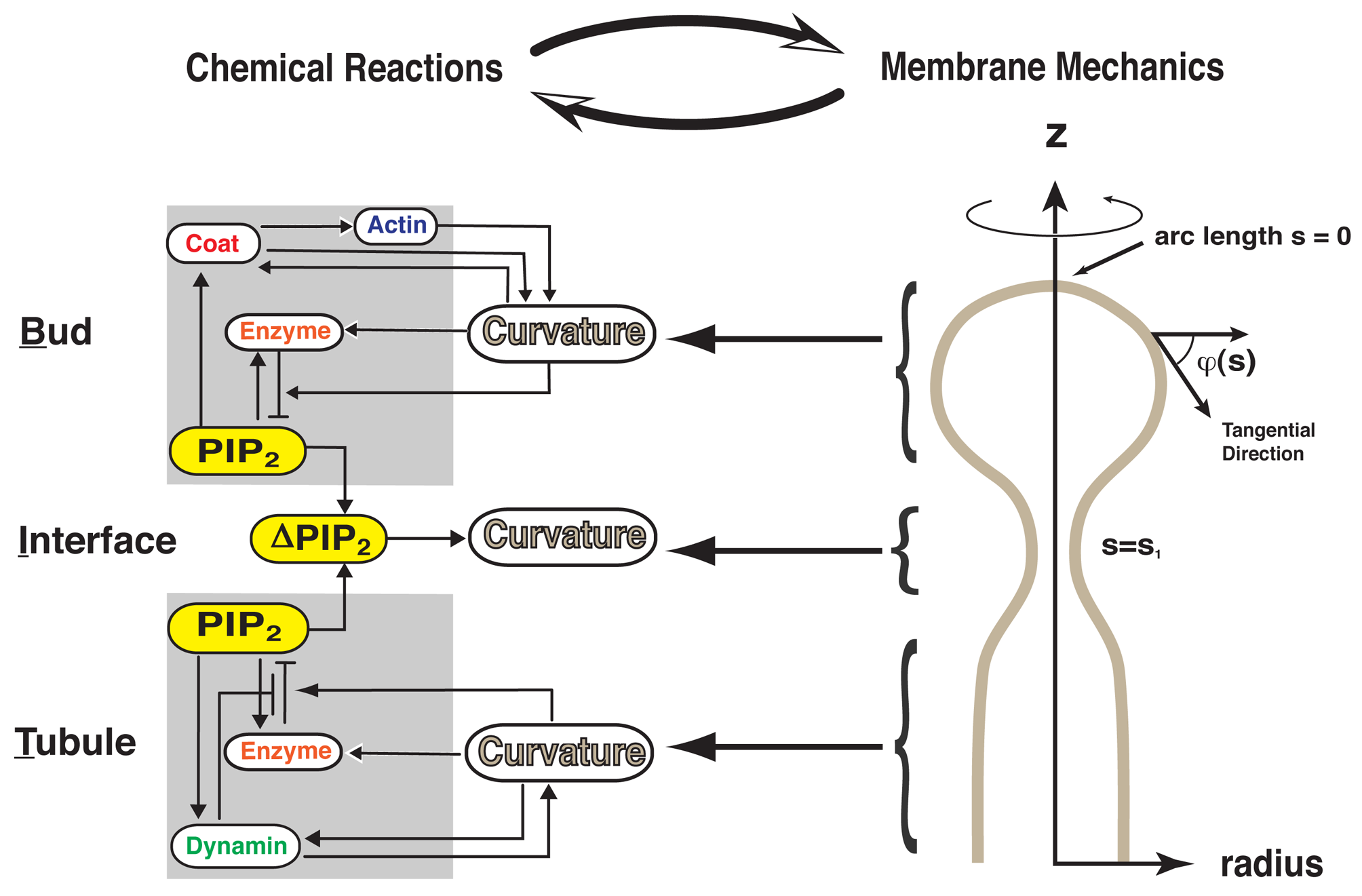

Supplement: Figure S5 — The interaction diagram amongst the functional modules in mammalian endocytosis (please see Section F in Protocol S1 for details). (0.45 MB TIF) [file pbio.1000204.s006.tif]

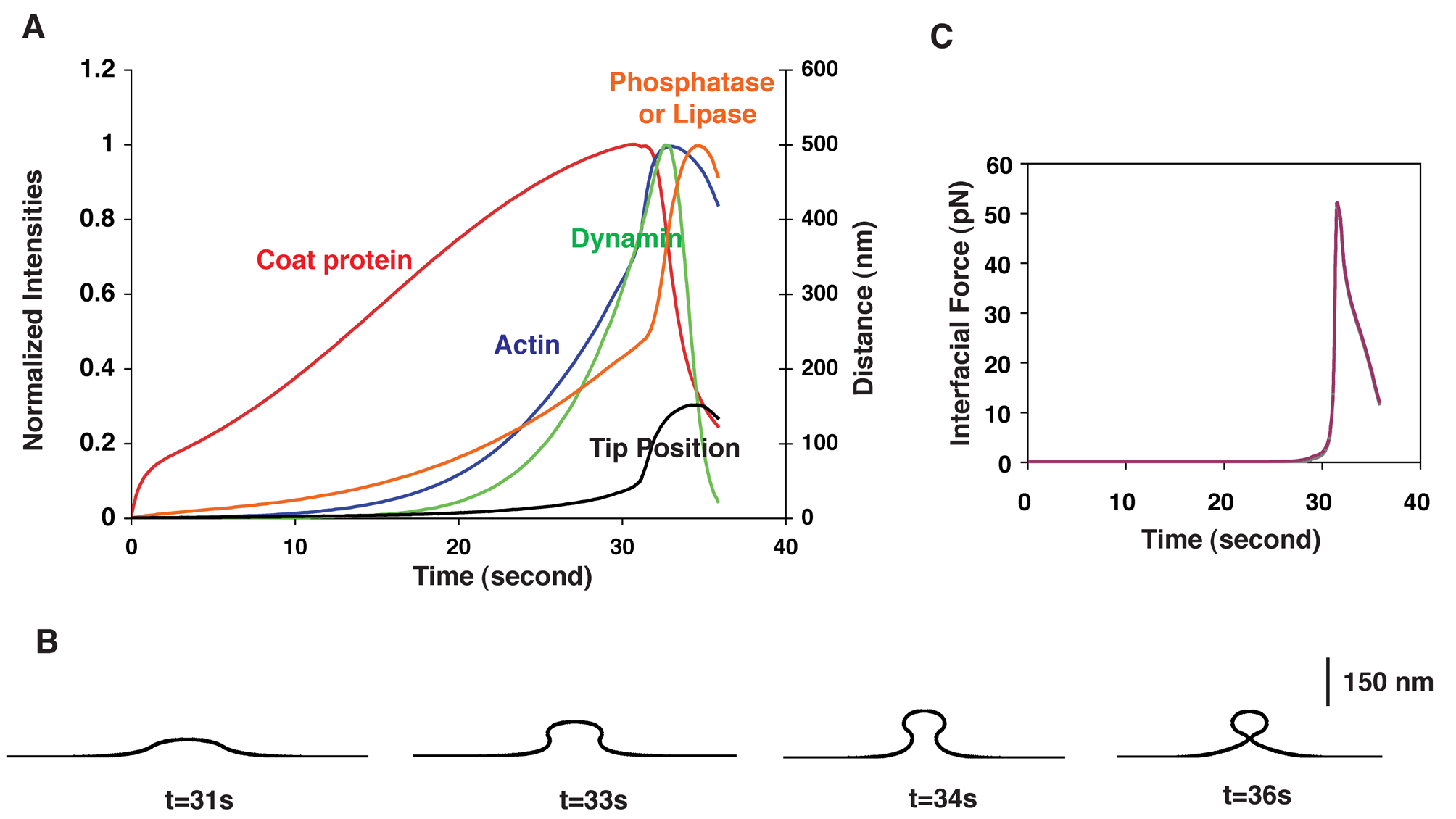

Supplement: Figure S6 — Endocytosis dynamics for mammalian cells. (A) Calculated time-lapse of the functional modules and the tip position of the endocytic membrane. (B) Snapshots of the calculated endocytic membrane shape changes. (C) The development of interfacial force over time. In contrast to budding yeast, the recruitment rate of dynamin is independent of actin and is much faster: we take it to be 4.0/s; actin polymerization and depolymerization rates are slowed down by 2-folds (22.5 nm/s and 15 nm/s, respectively). If not otherwise specified, the other parameters in this modified model are the same as those for yeast endocytosis (please see Section F in Protocol S1 for details). (0.37 MB TIF) [file pbio.1000204.s007.tif]

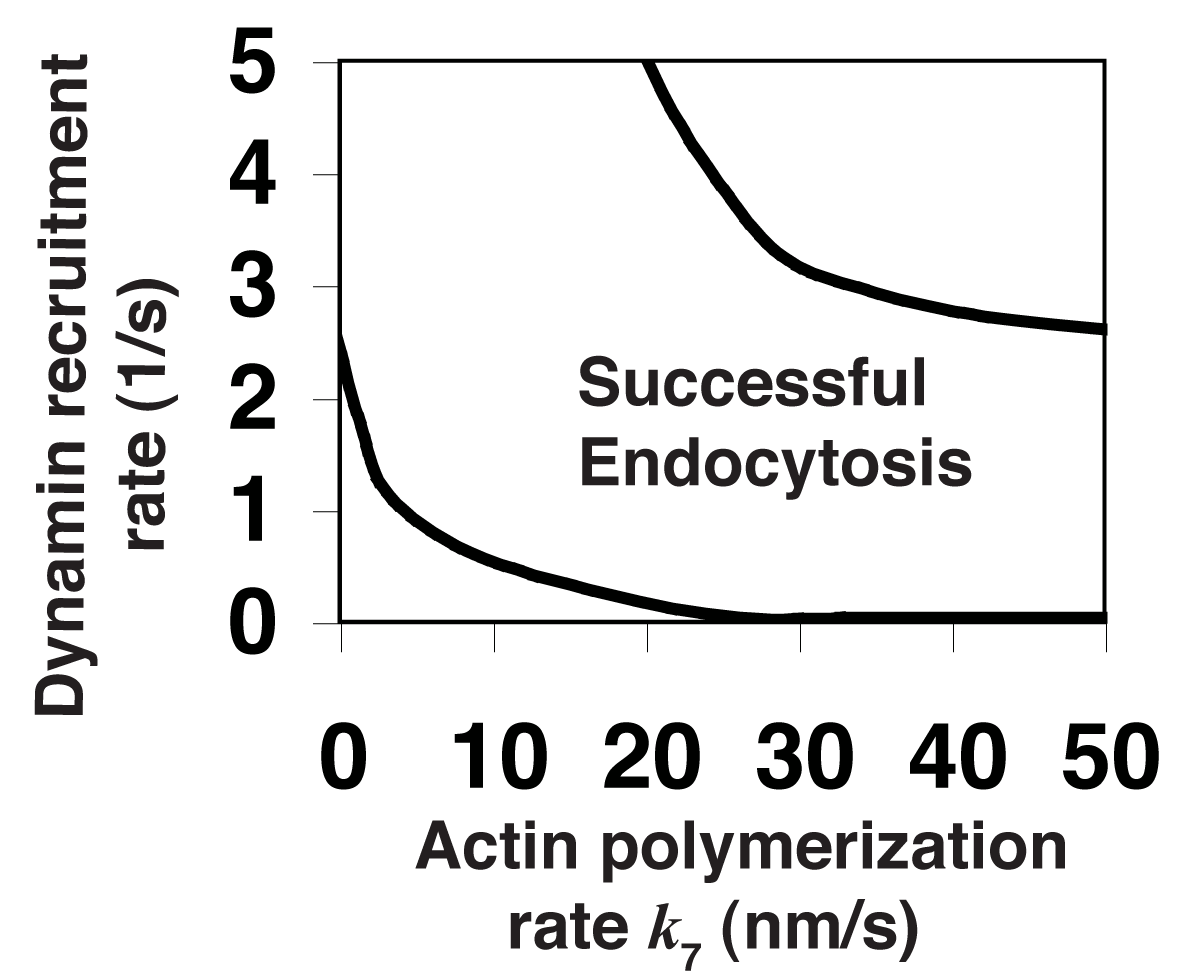

Supplement: Figure S7 — Phase diagram for mammalian endocytosis characterized by actin polymerization rate and the recruitment rate of dynamin. Note that there is a threshold value of dynamin recruitment rate ∼0.1/s, only above which endocytosis can be successful. Due to the resolution of the scales in y-axis, it is not shown here (please see Section F in Protocol S1 for details). (0.11 MB TIF) [file pbio.1000204.s008.tif]

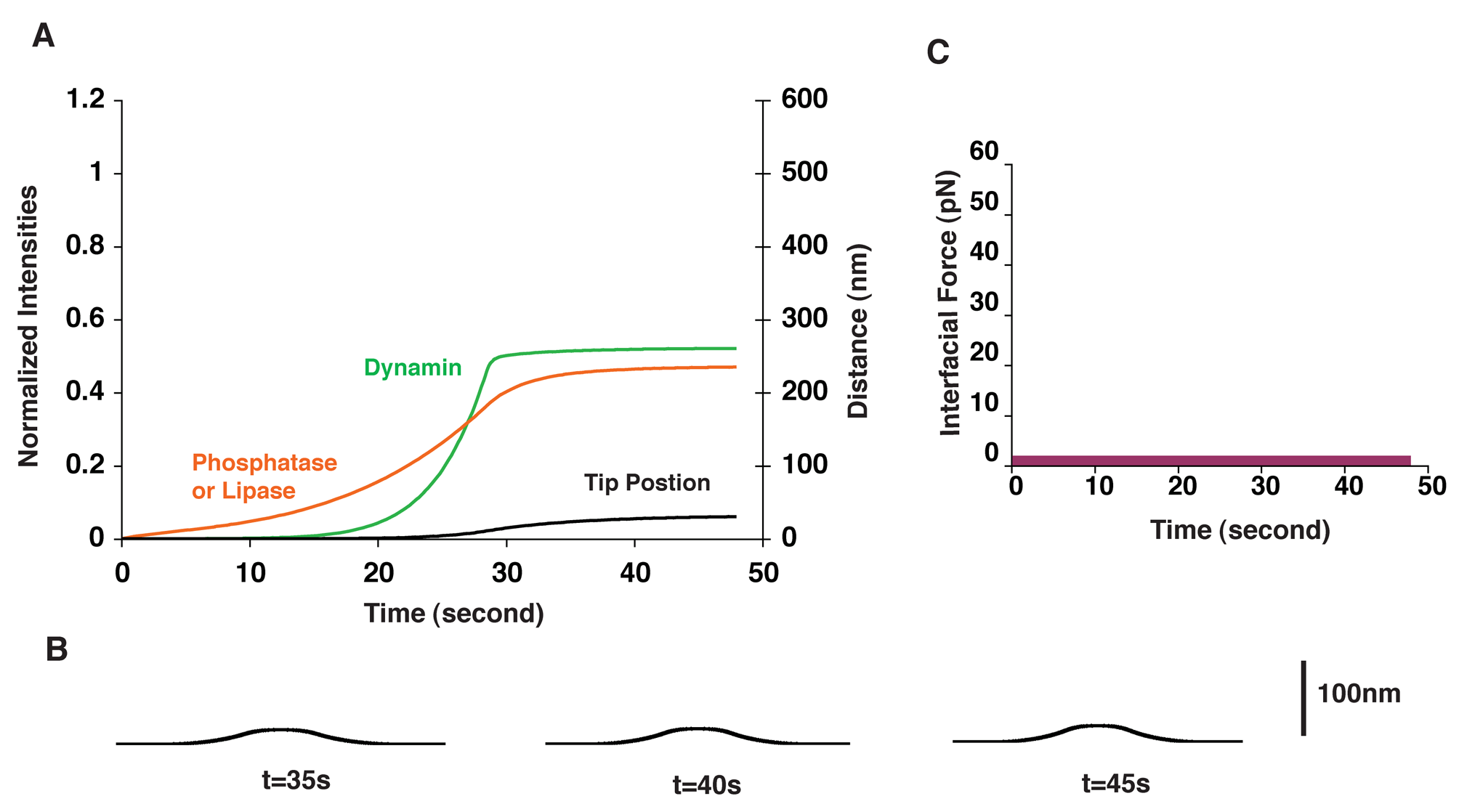

Supplement: Figure S8 — Predicted clathrin knock phenotype in mammalian endocytosis. (A) Calculated time-lapse of the functional modules and the tip position of the endocytic membrane. (B) Snapshots of the calculated endocytic membrane shape changes. (C) The development of interfacial force over time. Here the recruitment rate of the coat protein at the bud is taken to be zero. The intensities of the proteins in (A) are normalized relative to those in Figure S4 (please see Section F in Protocol S1 for details). (0.25 MB TIF) [file pbio.1000204.s009.tif]

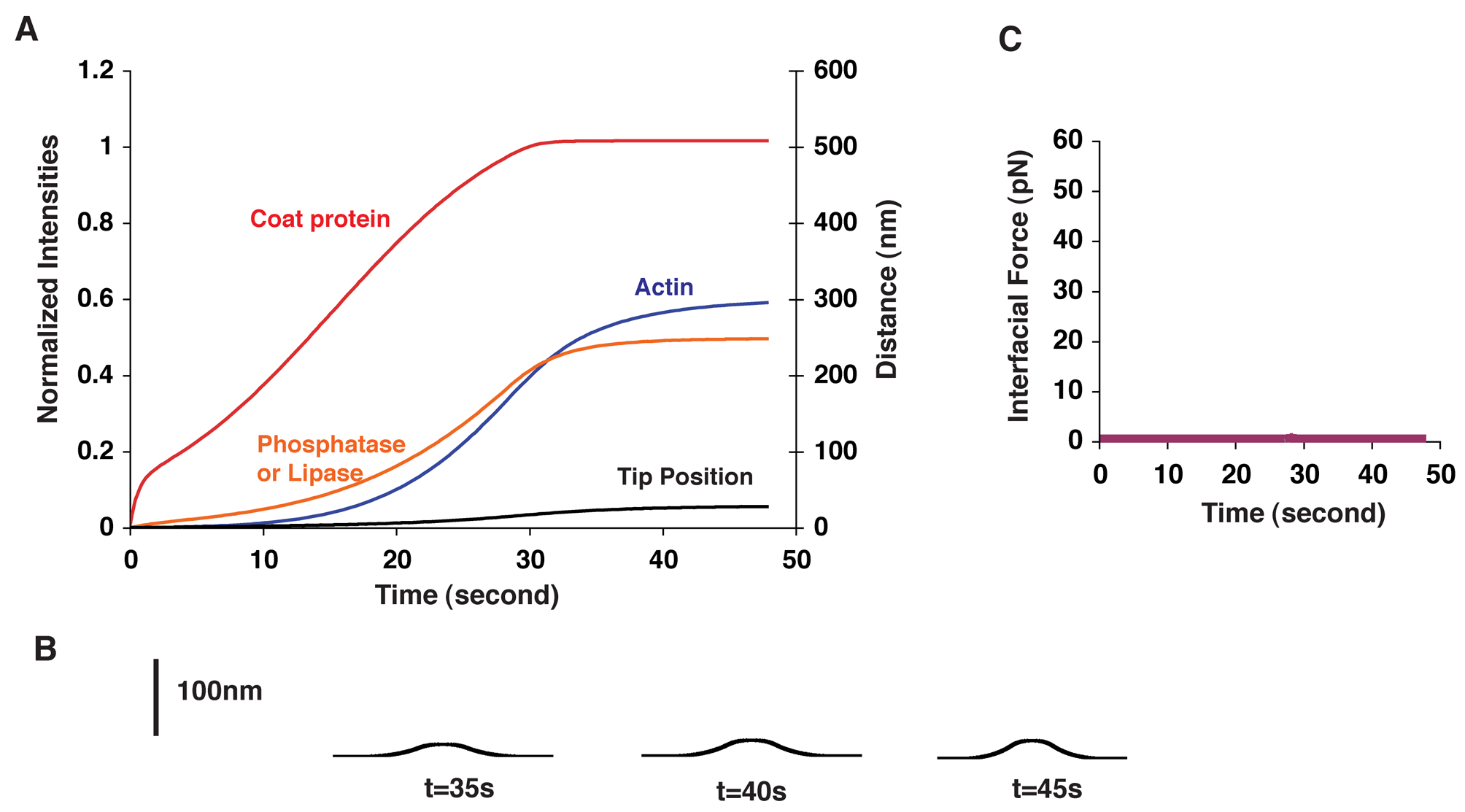

Supplement: Figure S9 — Predicted dynamin knockout phenotype in mammalian endocytosis. (A) Calculated time-lapse of the functional modules and the tip position of the endocytic membrane. (B) Snapshots of the calculated endocytic membrane shape changes. (C) The development of interfacial force over time. Here the dynamin recruitment rate is taken to be zero (please see Section F in Protocol S1 for details). (0.28 MB TIF) [file pbio.1000204.s010.tif]
